# A 25-Year Retrospective of Health IT Infrastructure Building: The Example of the Catalonia Region

**DOI:** 10.2196/58933

**Published:** 2024-11-18

**Authors:** Jordi Piera-Jiménez, Gerard Carot-Sans, Marina Ramiro-Pareta, Maria Mercedes Nogueras, Júlia Folguera-Profitós, Pepi Ródenas, Alba Jiménez-Rueda, Thais de Pando Navarro, Josep Antoni Mira Palacios, Joan Carles Fajardo, Joan Ustrell Campillo, Emili Vela, David Monterde, Damià Valero-Bover, Tara Bonet, Guillermo Tarrasó-Urios, Roser Cantenys-Sabà, Pau Fabregat-Fabregat, Beatriz Gómez Oliveros, Jesús Berdún, Xabier Michelena, Isaac Cano, Rubèn González-Colom, Josep Roca, Oscar Solans, Caridad Pontes, Pol Pérez-Sust

**Affiliations:** 1 Catalan Health Service Barcelona Spain; 2 Digitalization for the Sustainability of the Healthcare System (DS3) research group Barcelona Spain; 3 Faculty of Informatics, Multimedia and Telecommunications Universitat Oberta de Catalunya Barcelona Spain; 4 Agency of Health Quality and Assessment of Catalonia Barcelona Spain; 5 Center for Telecommunications and Information Technologies Barcelona Spain; 6 Catalan Institute of Health Barcelona Spain; 7 Hospital de la Santa Creu i Sant Pau Barcelona Spain; 8 Rheumatology Unit Vall d'Hebron Hospital Universitari Vall d'Hebron Barcelona Hospital Campus Barcelona Spain; 9 Hospital Clínic de Barcelona Barcelona Spain; 10 Institut d'Investigacions Biomèdiques August Pi I Sunyer (IDIBAPS) Barcelona Spain; 11 Universitat de Barcelona Barcelona Spain; 12 Universitat Autònoma de Barcelona Barcelona Spain; 13 Catalan Department of Health Barcelona Spain

**Keywords:** health ITs, eHealth, integrated care, open platforms, interoperability, Catalonia, digitalization, health care structure, health care delivery, integrated pathway, integrated treatment plan, process management

## Abstract

Over the past decades, health care systems have significantly evolved due to aging populations, chronic diseases, and higher-quality care expectations. Concurrently with the added health care needs, information and communications technology advancements have transformed health care delivery. Technologies such as telemedicine, electronic health records, and mobile health apps promise enhanced accessibility, efficiency, and patient outcomes, leading to more personalized, data-driven care. However, organizational, political, and cultural barriers and the fragmented approach to health information management are challenging the integration of these technologies to effectively support health care delivery. This fragmentation collides with the need for integrated care pathways that focus on holistic health and wellness. Catalonia (northeast Spain), a region of 8 million people with universal health care coverage and a single public health insurer but highly heterogeneous health care service providers, has experienced outstanding digitalization and integration of health information over the past 25 years, when the first transition from paper to digital support occurred. This Viewpoint describes the implementation of health ITs at a system level, discusses the hits and misses encountered in this journey, and frames this regional implementation within the global context. We present the architectures and use trends of the health information platforms over time. This provides insightful information that can be used by other systems worldwide in the never-ending transformation of health care structure and services.

## Introduction

The past 25 years have witnessed a progressive shift in the health care needs of the population. Factors such as aging, increased prevalence of chronic diseases, and heightened expectations for quality care have driven this evolution. The fast-paced changes in health care needs require an integrated and comprehensive approach that addresses the multifaceted health requirements of individuals [1].

Worldwide, health care systems have been continuously looking to improve the quality and efficiency of health care delivery. The late 1990s and early 2000s saw a shift from mechanistic, top-down approaches with minimal impact on outcomes to dynamic models recognizing health care as a complex adaptive system with a growing emphasis on multidisciplinary care, partnership working, and disease management [2]. Population-based approaches emerged, targeting specific groups such as older adults and integrating social care, education, and housing to address broader determinants of health [3-7].

In the last decade, the focus on integrated care has further evolved with advancements in technology and global trends such as advances in precision medicine. Strengthening primary care and moving toward a whole-of-system approach that links different levels of the health system has become crucial [8-10]. Functional integration, involving enhanced communication, improved care coordination, and robust clinical information flows, supports integrated care partnerships [11,12]. Initiatives such as accountable care organizations in the United States, where groups of health care providers work together to improve quality and reduce costs for Medicare patients by coordinating care and sharing in the savings achieved, are a good example of this [13-15]. Furthermore, the use of digital tools and health information systems has facilitated better care coordination and patient management, leading to value-based care models that incentivize quality and outcomes [16]. With this idea in mind, the National Health Service in the United Kingdom has recently launched the integrated care systems (ICSs) initiative to better coordinate services across health and social care, aiming for more efficient, patient-centered delivery [17]. Another example of this is the Dutch vision document on network medicine, which emphasizes the importance of connected care networks to address complex patient needs comprehensively [18]. The United States also emphasizes patient-centered medical homes and patient-centered medical neighborhoods to foster comprehensive, patient-focused care [19,20].

In parallel, there has been an unprecedented evolution in information and communications technologies across all life domains—including health care—with a potential to revolutionize health care delivery. Innovations in telemedicine, electronic health records (EHRs), digital diagnostic tools, and mobile health apps exemplify how technology can enhance health care accessibility, efficiency, and patient outcomes [21,22]. These developments suggest a future in which health care is more personalized, data driven, and accessible than ever before.

**Figure 1:**
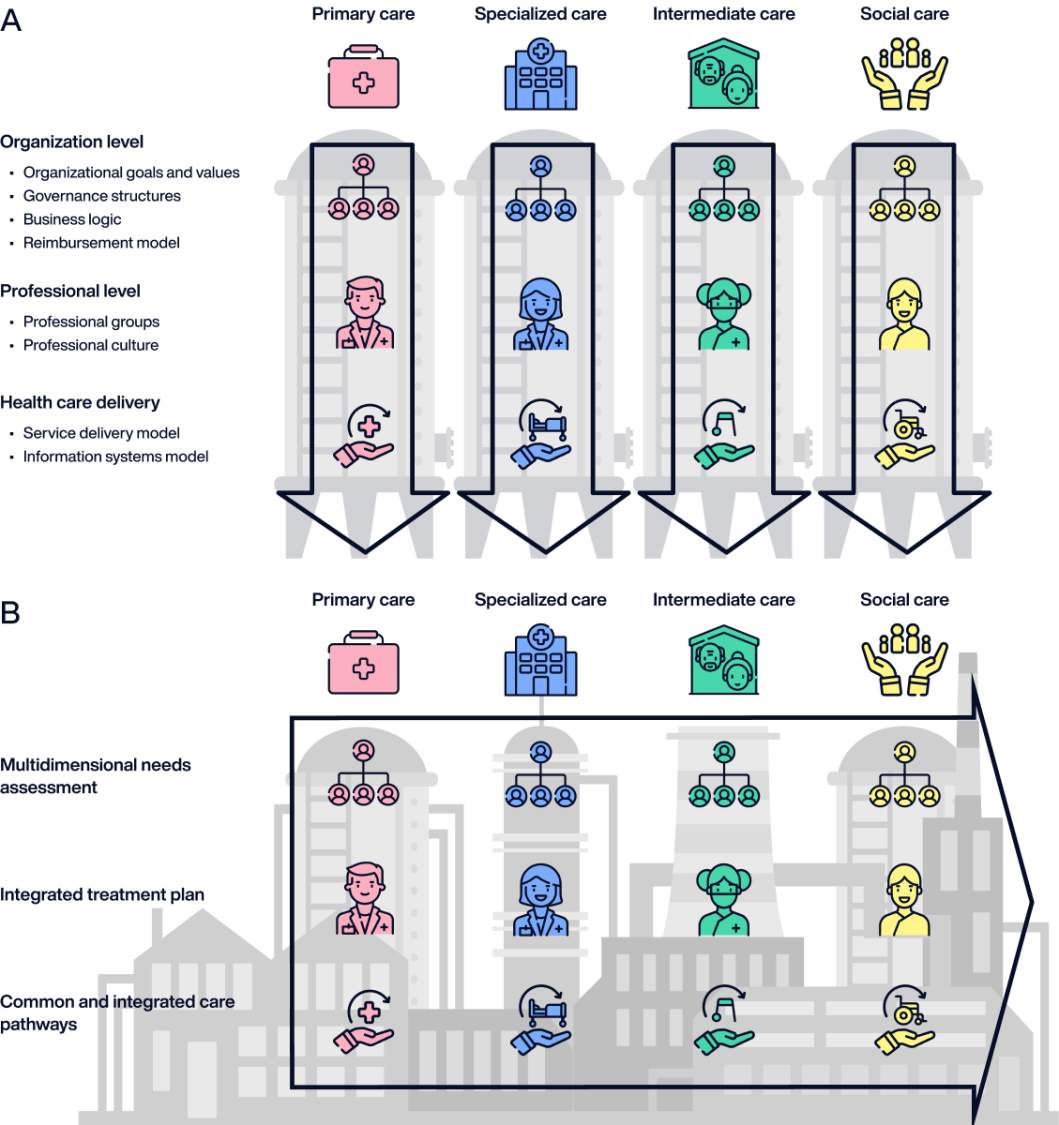
Paradigm shift in the health care delivery model—from vertical siloed care (A) to collaborative care (B; adapted from the work by Amelung et al [25]).

Despite these technological advancements, organizational, political, and cultural constraints have hampered the effective integration of new information and communication technology solutions into health systems worldwide [23]. A notable issue identified elsewhere is the prevailing siloed approach to health care information management [16,24]. Most health care organizations still operate with fragmented systems, hindering the seamless collection, storage, and sharing of health information [16]. This compartmentalization jeopardizes the deployment of resilient health care models that can deliver person-centered care in an increasingly demanding context [25] (Figure 1 [25]).

This Viewpoint showcases the development and implementation of health ITs (HITs) in Catalonia over the past 25 years and frames them in the global context of digital health transformation. Owing to the early adoption of digital solutions, Catalonia represents a useful case study to describe the multiple interventions that can be implemented in health information systems at the country level and appraise the pros and cons of each of them. Therefore, the aim of this Viewpoint was to provide insightful information to countries that are endeavoring their journey through the digitalization of HITs. After describing the structural context of the Catalan health system and providing a chronological overview of the digital transformation of HITs, we thoroughly describe each of the HIT solutions, grouped according to their overall purpose into data integration for clinical support, patient-system interaction, and health care management and public health.

## Context and Setting

Catalonia is an autonomous community in Spain with a population of approximately 8 million people distributed across an area of 32,114 km^2^, primarily concentrated in urban areas. As in many other countries in Europe [26], the Catalan population has experienced an aging shift over the past few decades [27]. The health care system in Catalonia is integrated into the Spanish National Health System (NHS), primarily funded through taxation, and provides universal health care coverage to the entire population. Services under the Spanish NHS are generally provided free of charge at the point of delivery [28]. Nevertheless, according to the Catalan Health Survey, 32.5% of the Catalan citizens have a private voluntary insurance, mostly to cover complementary services to those under the statutory health system, such as reduced waiting times for specialized care and access to services that are restricted within the public benefits package [29].

In Catalonia, health care services are delivered through a network of providers that operate at four levels of care: (1) primary care (369 facilities); (2) specialized care, including public and semiprivate (publicly subsidized) hospitals (69 facilities); (3) intermediate and long-term care (96 facilities); and (4) mental health care (42 facilities) [30]. Health care in Catalonia is organized into 10 health regions, each with its network of facilities, which enables tailoring health care services to the demographic characteristics of each area. Outpatient drugs are prescribed by physicians, either general practitioners (GPs) or specialists providing care through public coverage, and dispensed at private community pharmacies based on a copayment system [28] (Multimedia Appendix 1).

Catalonia exhibits a high degree of digital advancement, as evidenced by its 58% digital maturity index (a measure of the region’s effective use of digital technologies to improve processes and services), a high satisfaction rate among e–government service users, and an estimated internet penetration rate of >93% [31].

## The Never-Ending Metamorphosis

### Overview

According to Vial [32], digital transformation can be defined as a process that aims to improve an entity by triggering “significant changes to its properties through combinations of information, computing, communication, and connectivity technologies.” Digital transformation processes embody the dynamic and ongoing evolution of organizational operations, culture, and end-user experiences through the integration of digital technology [33]. This journey is inherently complex and multifaceted, reflecting the continuous innovation and adaptation that characterize the digital era. Digital transformations are not linear or predictable; instead, they are iterative, requiring constant adaptation to each cycle of technological advancement in response to new and changing market demands [34].

**Figure 2:**
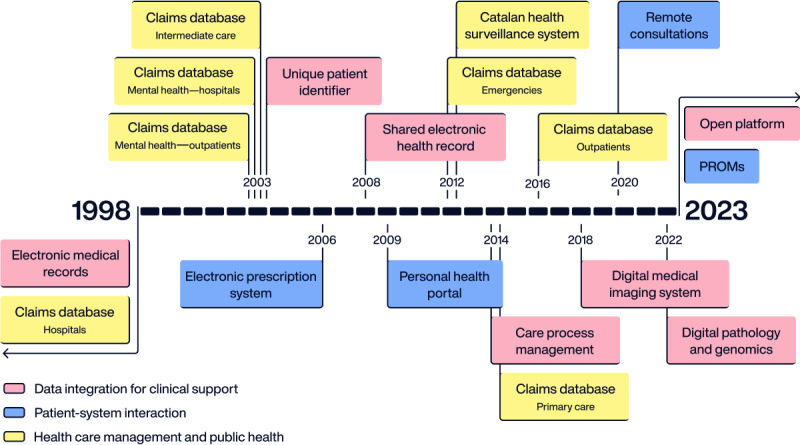
Transformation road map in Catalonia, including the different systems comprising the Catalan eHealth platform for the period of 1998 to 2023. PROM: patient-reported outcome measure.

In the past 25 years, the landscape of HIT in Catalonia has been progressively reshaped through a transformation process that is still ongoing and started right after the first digitalization of medical records in the late 90s. Even though the health care sector has been slower than other industries in the adoption of technology, much has changed over the past 25 years. This section dives into the metamorphosis of the health care sector in Catalonia, catalyzed by the adoption of HIT within the period ranging from 1998 to 2023. First, we present the state of the health care sector at the beginning of the period and systematically describe the transformation journey and the drivers of transformation leading to the comprehensive eHealth platform (Figure 2).

Although the digital transformation milestones occurred subsequently on a timeline, for clarity and consistency, we present the results of this retrospective grouped into three categories of HIT infrastructures aimed at (1) integrating data to support clinical decision-making, (2) improving the interaction between patients and the health care system, and (3) health care management and public health.

Briefly, the journey kickoff was the introduction of administrative systems designed to handle patient appointments, billing, and other clerical tasks. The adoption of electronic medical records (EMRs) followed this first step, endeavoring an implementation process that was fostered by the Spanish entry into the European Union in 1986 and spanned the 90s. These early iterations of EMRs consisted of digitalizing patient records within specific health care settings, with limited—if not missing—data sharing capabilities. This marks the initial stage of the analyzed period, characterized by stand-alone solutions designed for serving single health care provider organizations. While these systems represented a significant step forward in managing patient information digitally, their isolated nature posed substantial challenges for the continuity of care.

### Data Integration for Clinical Support

#### Overview

Providing health care professionals with comprehensive information on patients has been for years the backbone of the digitalization of health information systems. For this information to be useful and safe, it should integrate the health data of a given patient regardless of the health care setting or place where they were created [35]. The universal health care coverage, paired with a unique patient identification number, was a mainstay for this approach. However, our health care landscape, with many providers with different health information systems, hampered the full integration of all patients’ data and required implementing alternative solutions, such as shared health records or federated platforms.

#### Unique Patient Identifiers

In Spain, the cornerstone for integrating health information was laid in 2003 with the introduction of the individual health card (in Catalan, Targeta Sanitaria Individual), associated with a unique patient identifier code and established as a requisite for nonurgent health care access within the Spanish NHS [36]. This initiative also led to the creation of a protected database that collects essential information about the insured population. In Catalonia, this registry was named Central Registry of Persons Insured (in Catalan, Registre Central d’Assegurats) and contains essential demographic information needed for assigning health care resources to all Catalan inhabitants.

The interoperability of the unique patient identifier system is supported by a robust technical infrastructure that includes a communications network, standards for devices and cards, and an XML-based messaging system. This design allows for the fluid exchange of health information between regions, guaranteeing that users receive care and their prescriptions across Spain, thus reinforcing Spanish NHS cohesion and quality [37].

Catalonia, recognizing the potential of unique patient identifiers, has been transitioning toward a master patient index since 2020. This system aims to track all individuals receiving care within the region extending beyond Catalan and Spanish inhabitants (ie, including tourists and other people being served by the public health services). Built upon the Fast Healthcare Interoperability Resources (FHIR) standard, the master patient index allows for flexible data management and accurate patient matching across different providers within the ecosystem.

#### Shared EHR

At the center of the Catalan eHealth platform is the shared EHR of Catalonia (locally known as Història Clínica Compartida de Catalunya in Catalan), established in 2008. The shared EHR was founded on the Catalan Law 21/2000 about information rights, patient autonomy, and clinical documentation, which established “the objective to advance towards a unique medical record per patient” [38]. The main objective of this record is to improve the quality of health care delivery by providing an instrument that aids health care professionals in clinical decision-making by enabling access to relevant health information across Catalan health care providers [39].

From a technical perspective, the shared EHR was designed considering the fragmentation of health information systems in the Catalan health care ecosystem. Therefore, it was built by pooling together information collected by multiple EMRs (ie, 30 in total) in a central repository. Locally, information is collected following different standards but normalized according to international messaging and semantic interoperability standards (ie, Health Level 7 Clinical Document Architecture [HL7-CDA], Systemized Nomenclature of Medicine–Clinical Terms [SNOMED-CT], and Local Observation Identifiers Names and Codes [LOINC]) when transferred to the central record. The unique patient identifier works as a link for connecting information from different sources to a given patient. Service provider organizations can consume push-and-pull web services, allowing them to seamlessly integrate the information contained within their clinical workstations. For those who do not have the capabilities to integrate the information into their EMRs, a web-based viewer is provided.

From a security perspective, the record is only accessible by the homologated public service provider organizations through the virtual private network of the Catalan public health system. Security assertion markup language tokens are provided to service providers, and a 3-level security schema is followed (ie, patient identification, provider identification, and type of information), ensuring full traceability and legal compliance.

Since its inception, the shared EHR has been increasing the quantity and quality of the information collected, moving from semistructured documents into more granular information. Table S1 in Multimedia Appendix 1 provides an overview of the type of information contained and data volume. On December 31, 2023, the storage capacity of the database was 60 TB (including auditing). This system allows health care professionals to track their patients throughout the health care system, reviewing reports generated in other health care facilities (eg, due to emergency consultations, specialized interventions and tests, or regular consultations done in health care centers other than the regular one). Although the benefits of this record have not been formally assessed, it was deployed with the objective to reduce duplicated assessments, aid in diagnosis, and improve the continuity of care across the Catalan health care ecosystem.

#### Care Process Management

The care process management platform (locally known as Integrador de Serveis de Salut in Catalan), established in 2014, aimed to revolutionize the way in which health and social care services are coordinated and delivered, building on the principle that person-centered health care delivery often extends beyond the edges of a single organization [40,41]. The platform was designed by the Catalan Health Service, which maintains the specifications, and is provided free of charge to all service provider organizations within the public health care ecosystem in Catalonia. By providing a common language and configurable workflows, the platform enables diverse health care entities to interact efficiently, share critical information, and deliver coordinated care in a standardized way.

Functionally, the care process management platform targets key areas to improve the health care experience for all stakeholders. Citizens are expected to experience smoother transitions when crossing organizational boundaries, reduced duplicated tests, and improved patient safety and security. Health care providers benefit from improved interoperability and integration with existing information systems, facilitating better care delivery and enabling a holistic view of patient health. Health services are expected to see enhanced resource planning, reduced waiting times, and an improved use of resources.

**Figure 3:**
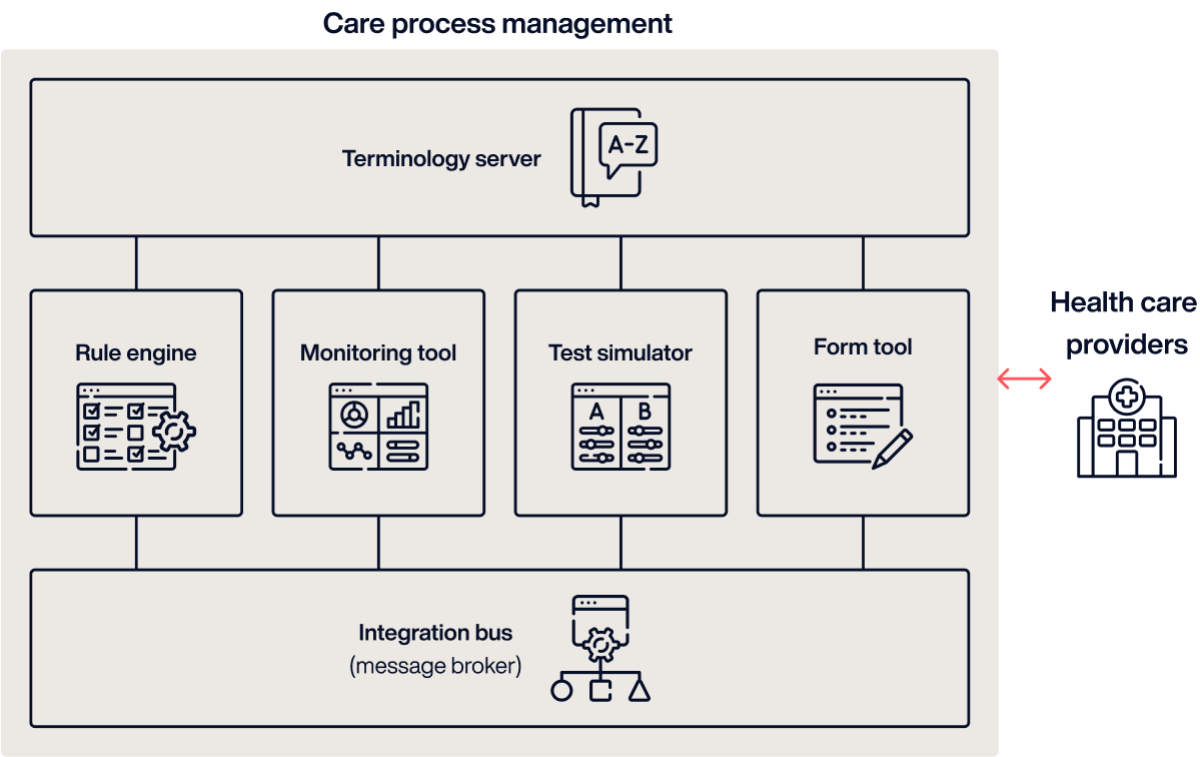
Architecture and main components of the care process management platform.

Technically, the platform is built on a foundation of interoperability and uses a range of technologies to ensure seamless communication and data sharing across different systems and stakeholders. Key technical features include a message broker for centralized messaging, rule engine for business logic, monitoring of tools for real-time data tracking, a configurable form tool, and a terminology server to standardize health care terminology across systems (Figure 3).

Interoperability is achieved through three main layers: (1) semantic, ensuring consistent meaning and use of data; (2) syntactic, standardizing the structure of communication through Health Level Seven messaging; and (3) technical, defining the communication channels and standards for data exchange. This layered approach ensures that information flows smoothly and meaningfully across different health care and social services, supporting better decision-making and facilitating continuous and integrated care.

The implementation of care process management platform spans various health care settings, including mental health centers, primary and specialized care, social care facilities, and emergency services. However, the model is mainly used as a referral system, and to date, it does not allow for setting integrated care workflows across providers.

#### Federated Platforms

While initially relying on a decentralized system (ie, every provider having its own infrastructure), challenges such as data fragmentation and barriers to innovation prompted a shift toward a federated model. In this model, the Catalan eHealth platform provides a central infrastructure to which all service provider organizations connect, avoiding the need for them to support big storage facilities, particularly for managing structured data such as medical images, pathology, and genomics. This federated approach overcomes important hurdles for data sharing and interoperability, such as diagnosis and treatment delay, and, at the same time, allows for economies of scale in terms of costs of data storage and security. In the future, this model will likely facilitate implementing machine learning algorithms for clinical decision support across the region due to higher volumes of data stored in a single repository.

**Figure 4:**
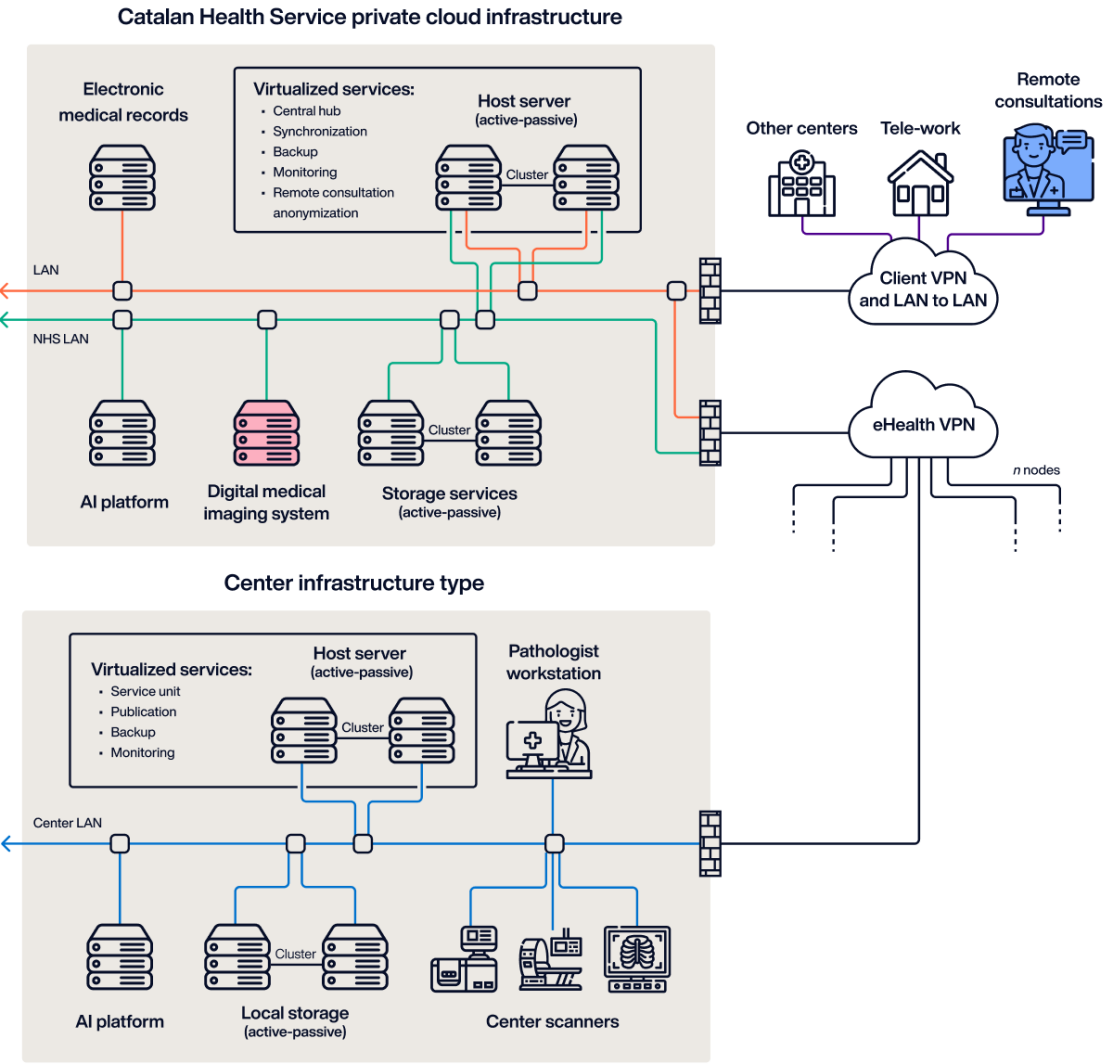
Example architecture of a federated information platform—Digital Medical Imaging System of Catalonia and Digital Pathology Transformation of the Catalan Health Institute. AI: artificial intelligence; LAN: local area network; NHS: National Health Service; VPN: virtual private network.

Both the Digital Medical Imaging System of Catalonia (SIMDCAT) and the Digital Pathology Transformation of the Catalan Health Institute initiatives [42] are prime examples of how digital tools and platforms are integrated into this centralized model to improve the management and accessibility of medical images and pathology data (Figure 4). Following the same strategy, Catalonia is currently working on the construction of a federated network for genomics, starting with precision oncology [43]. The strategic focus on precision oncology aligns with the Catalan Health Service’s investment in specific genomic panels for primary use, underscoring the importance of robust tracking for all interventions in this specialized area. This prioritization reflects an understanding of opportunity cost in health care resource allocation; by focusing on precision oncology, Catalonia is maximizing the potential benefits of its investment in genomic technologies while acknowledging that resources directed there cannot be simultaneously invested in other areas. This approach allows for targeted implementation and evaluation of the federated genomics network in a high-impact area before potential expansion to other genomic applications.

For reference, the current storage capacity of the Digital Pathology Transformation of the Catalan Health Institute amounts to 2.4 PB, corresponding to 37 million pathology exams (approximately 1 PB per year). Correspondingly, the SIMDCAT platform contains 3.7 PB of 118 diagnostic imaging technique examinations. Results from computed tomography and radiography, digital radiography, ultrasound, electrocardiography, mammography, and magnetic resonance account for 90% of the total examinations. Table S2 in Multimedia Appendix 1 provides the entire list of diagnostic imaging techniques and their volume currently stored in the SIMDCAT platform.

The improvement of communications in the region and the deployment of cloud computing have been the main technological driver for this transformation, which not only improves the operational aspects of health care delivery but also sets the stage for future innovations that can further enhance the effectiveness and efficiency of health care services in the region.

### Patient-System Interaction

#### Overview

The shift from diagnosis-centered to person-centered care and the increasing involvement of patients in their care are among the most important changes in health care routines in the past few years and are acknowledged as high-quality practices for future health care systems [44,45]. This approach requires allowing patients access to their own health care data, thus adding an additional challenge for health information infrastructures.

The drug prescription system and the personal health portal were significant breakthroughs in our digitalization journey that enhanced the interaction between patients and the health care system. The consolidation of these 2 milestones allowed us to build more innovative tools, such as remote consultations and patient-reported outcome measures (PROMs).

#### Personal Health Portal

With the development of the My health platform in 2009, Catalonia was among the leading regions in early implementation of patient health portals [46]. My health is a comprehensive tool that allows citizens to access and manage their personal health information. Users of this platform have the convenience of viewing their medical records web-based, which includes details such as physicians’ notes, test results, medications, and information about their vaccinations and allergies. One of the key services of the platform is the ability to manage appointments with health care professionals. Users can schedule, reschedule, or cancel their appointments through the platform, making health care more accessible and user-friendly [47].

The platform also provides users with important information about their health plans, including what services and coverage they have as well as test results, including laboratory assessments and reports of diagnosis techniques. This can be particularly helpful in understanding and navigating the health care system. In addition, My health includes a variety of health resources and educational materials, offering guidance and information on preventive care and general health maintenance.

Technologically, the platform is a subset of all the objective information in the shared EHR of Catalonia. This integration ensures that users have a comprehensive and up-to-date view of their health record and current treatments. This aspect of the platform underscores the broader move toward eHealth solutions that aim to enhance the efficiency, personalization, and accessibility of health services.

Even though the My health platform was deployed in 2009, its use was low among the population. The COVID-19 pandemic was a great opportunity to boost its use by moving added-value services into it [48,49]. The addition of electronic prescriptions and sick leave forms, COVID-19 digital certificates and tests results, and immunizations made the number of registered users explode (increasing from 600,000 in March 2020 to 5.8 million in December 2023). Currently, a median of 376,000 users access the system on a daily basis.

#### Electronic Prescription System

Although originally developed for invoicing purposes, the electronic prescription system has dramatically changed patients’ routines, shifting from regular face-to-face visits to their GP to collect a handful of messy handwritten papers to structured medication plans accessible by the GPs, specialists, pharmacists, patients, and the public health insurer.

In Spain, drug prescription is tightly controlled, limiting the issue of one medication per prescription and up to 3 months of advanced prescriptions [50]. Initially, a paper-based system required patients to visit their GP quarterly to obtain multiple prescription forms for each long-term medication, which they would then take to pharmacists for dispensing, often receiving all 3 months’ supply at once. This handwritten process risked medication errors and made it difficult to monitor patient medication plans, increasing the chance of dangerous drug interactions, especially with the addition of acute medications. Moreover, despite the advice to pick up medications monthly, many patients collected them all at once, leading to waste if medication plans changed.

**Figure 5:**
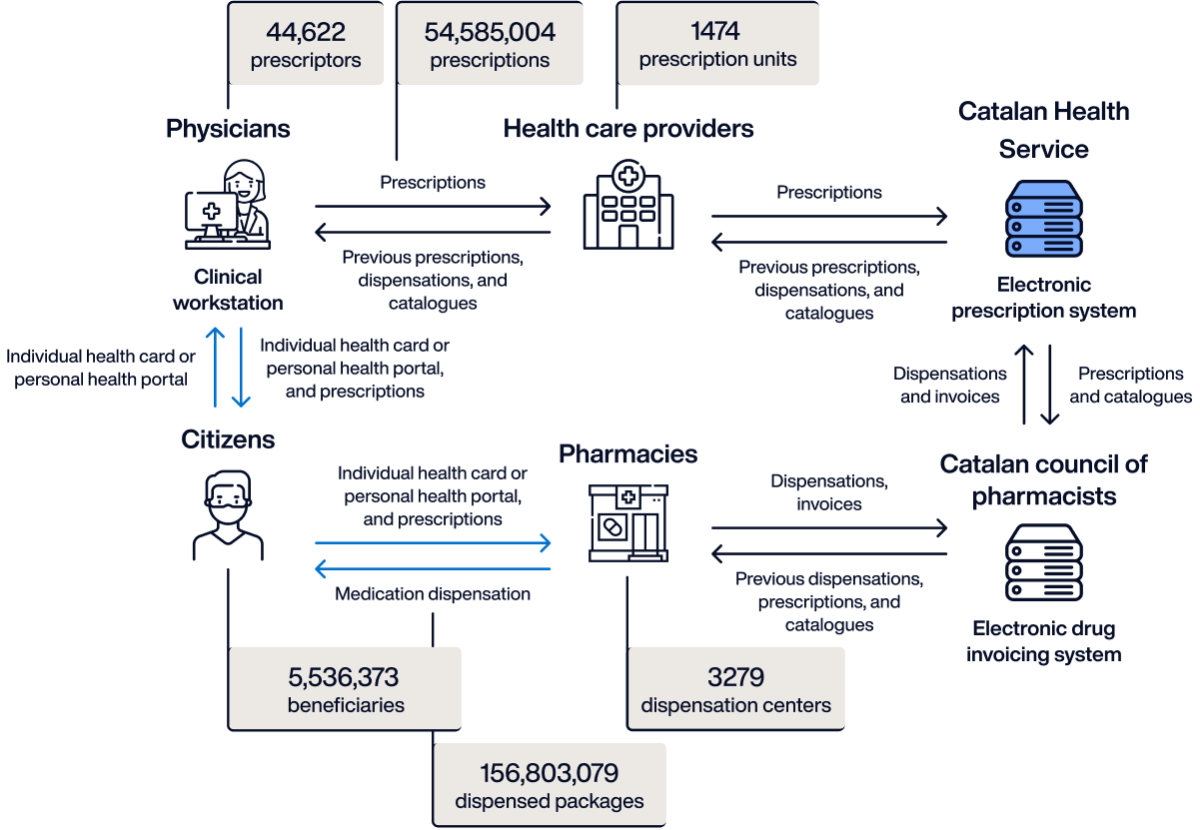
Information flow within the electronic prescription system and general use figures in 2023.

In the 2000s, the Catalan Health Service led the first deployment in Spain of a single electronic prescription system (locally known as Sistema Integrat de Recepta Electrònica in Catalan), aimed to be used for outpatient prescriptions across the entire public health care ecosystem in Catalonia. Through liaison with the Catalan College of Pharmacists, a mirrored platform was built to support dispensation and data collection from the 3200 community pharmacies for invoicing purposes. The College of Pharmacists worked to progressively harmonize the different versions of pharmacy management software to ensure connection and performance of the dispensation and invoicing process through the electronic prescription service (Figure 5). The process was piloted in 2006 and came into effect in 2007, reaching universal coverage 2 years later in 2009 [51-53].

The electronic prescription system allowed professionals to prescribe into a single, shared, and traceable medication plan and apply prescription support systems with basic checks for safety and drug interactions. The national regulation was updated to cover electronic prescription, allowing for the programming of successive monthly prescriptions for chronic medications for up to 1 year (automatically released to patients once a month), thus reducing the number of medical visits solely aimed at collecting new paper prescriptions while preventing patients from collecting an excess of packages in advance [50]. During the COVID-19 crisis, the electronic prescription system platform was also used for prescribing and invoicing antigen rapid-diagnosis tests done at community pharmacies for mass-testing strategies in schools and other community settings. The COVID-19 pandemic also encouraged ruling out paper for medication plans, with an estimate saving of 46 million paper sheets (ie, 600 tons of paper) [54]. The Catalan electronic prescription system is fully connected to those of the other Spanish regions and with the European ePrescription and eDispensation initiative [55].

Hospital medication has traditionally received less attention regarding information integration due to the localized activity and higher control by hospital care. However, outpatient medication prescribed from hospitals has growth, resulting in an increasingly high treatment burden and economic impact. In the mid-2010s, a dedicated registry (Registre de Pacients i Tractaments in Catalan) was created with a twofold purpose: to (1) keep a track record of the use and main clinical results of medications and (2) provide a tool for supporting patient-level invoicing of medication by hospitals. This registry has allowed for the assessment of quality and effectiveness for clinical and managerial purposes as well as the implementation of several outcome-based drug access agreements [56].

The next step in HIT related to drug prescription is to integrate all prescription information regardless of the setting (ie, in-hospital, outpatient, or community) into a single therapeutic plan that allows for safety surveillance and knowledge-based management of therapeutics. This strategy is being implemented within the Information Systems Master Plan of the Catalan Health Service [57].

#### Remote Consultations

In 2015, Catalonia witnessed an important transition to remote consultations, which began with the implementation of eConsultation, a digital platform that enables asynchronous communication between patients and health care professionals, facilitating health care continuity. eConsultation allows for 2-way asynchronous communication, enabling the exchange of messages, medical documents, reports, and test results between patients and health care providers [58,59]. This transition from traditional face-to-face interactions to a more flexible and efficient digital interface marks a significant evolution in health care delivery methods.

**Figure 6:**
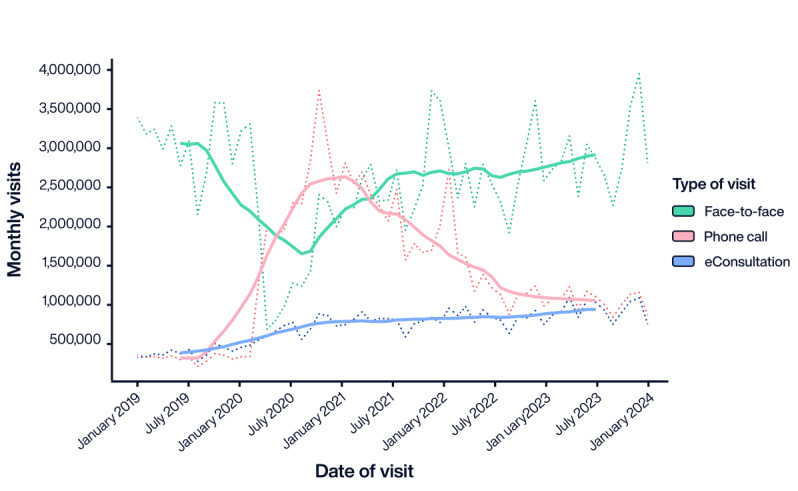
Monthly evolution of patient visits to health care professionals in Catalonia (January 2020-January 2024).

During the early stages of eConsultation implementation, this modality of asynchronous consultation was used by young patients, mostly women, with simple health issues [59]. Health care professionals, including physicians and nurses, mainly used eConsultation for consultative and managerial tasks [58]. The onset of the COVID-19 pandemic, which led to nationwide lockdowns dictated to contain the spread of SARS-CoV-2, catalyzed a significant uptick in eConsultation use, facilitating its broader acceptance and integration into the daily practices of health care professionals (Figure 6). The intensive use of eConsultation, including proactive patient communication, issue of sick leave certifications, prescription renewal, and addressing general medical inquiries remotely, shifted toward a hybrid model that melds traditional health care with telemedicine approaches and heralds a new era in health care delivery.

The COVID-19 crisis boosted not only asynchronous remote consultations but also video consultations. The accelerated and hasty implementation of remote consultations increased the risk of reducing the quality of health care delivery. Therefore, Catalonia endeavored an initiative (ie, the “Latitud” model) for an evidence-based implementation of remote consultations driven by a multidisciplinary team including experts from the health care, legal, and eHealth domains. Using an iterative process, which spanned May 2020 to June 2020, this team developed and disseminated evidence-based guidelines for health care professionals regarding the use of remote consultations [60]. The recommendations for professionals and organizations regarding the use of remote consultations are available in the Multimedia Appendix 1. This use framework is currently being assessed in a randomized controlled trial of synchronous remote consultations in the outpatient setting [61].

According to retrospective reports, remote consultations solved 88% of the cases, indicating a high capacity for reducing the work burden of health care centers [62]. This efficiency was particularly pervasive in the management of common issues such as test results, medical inquiries, and repeat prescriptions, which streamlined interactions and reduced unnecessary in-person visits. Of note, the reduction in face-to-face visits was concomitant with an increase in the demand for health care support due to its ease of access, potentially adding to the GPs’ workloads. Aside from the increased workload, limited digital literacy among some patient groups and the need for appropriately integrating telemedicine activities into professional workflows were identified as important challenges for the implementation and persistence of remote consultations in Catalonia [59].

#### Electronic Prescription of PROMs

PROMs are standardized questionnaires collecting patients’ health outcomes, directly provided by them with neither input from nor interpretation by health care professionals [63]. PROMs, often used for diagnosis or follow-up purposes, include a wide range of outcomes, such as symptoms, quality of life, psychological features, functional status, social issues, response to treatment, or patient satisfaction [64]. Electronic PROMs (ePROMs) allow for data collection via the internet on devices, increasing response rates. Furthermore, electronic collection of PROMs facilitates automated integration into EMRs, aiding clinical decision-making and promoting continuity of care. In recent years, ePROM prescription has been increasingly used in specialized care [65-68]; conversely, their use in primary care, where patient profiles are more heterogeneous, is still scarce [63,69,70].

**Figure 7:**
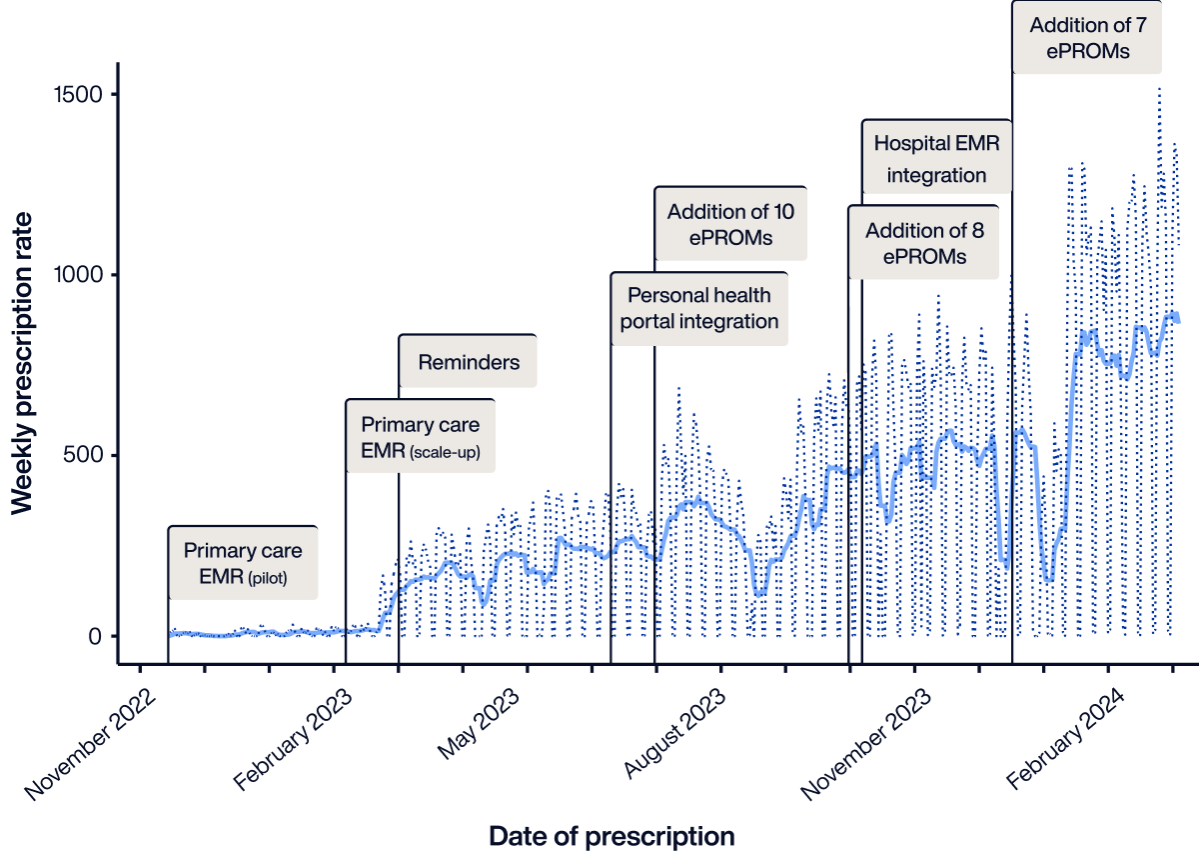
Progression of electronic patient-reported outcome measure (ePROM) prescription from pilot implementation to February 29, 2024. The dotted line shows the weekly crude rate, and the full line shows the 7-day moving average. The vertical lines represent key implementation events. EMR: electronic medical record.

In November 2022, an ePROM prescription module was implemented in the workstation of health care professionals at the system level. The design and implementation were driven by a panel of experts, including health care professionals and managers, and members of the internet-based PROM library in Spain (BiblioPRO) [71]. Implementation was carried out in three phases: (1) pilot implementation of 4 ePROMs in the primary care workstation of 11 preselected primary care centers, (2) scale-up across the whole primary care network, and (3) integration of the tool into the hospital’s workstations. Further enhancements of the tool were introduced in the months following implementation, including reminders to patients, integration into the personal health portal, integration into the shared EHR viewer, and addition of new ePROMs (Figure 7).

Currently, health care professionals in our system can prescribe ePROMs among a set of 29 ePROMs for several conditions (Table S3 in Multimedia Appendix 1). The prescription of ePROMs has increased since their implementation in the Catalan health care system (Figure 6). As of February 29, 2024, a total of 142,979 ePROMs had been prescribed by 6259 health care professionals, with 112,424 of these ePROMs being completed by patients, resulting in an average response rate of 78.7%. Therefore, in addition to supporting clinical decisions in routine care, ePROMs provide a significant amount of health information from the catchment population, paving the way for secondary use of data for benchmarking or research.

### Health Care Management and Public Health

#### Overview

Data collected with the primary purpose of health care delivery hold high potential for knowledge-based decision-making in nonclinical settings. Important activities for the quality of health, such as resource allocation, targeted public health or population-based assessment, and monitoring are currently based on the secondary use of clinical data. The single public insurer for health care services in Catalonia encouraged building registries and tools for benchmarking, invoicing, and monitoring the health status of the population.

#### Claims Registries

The earliest stages of the centralization of health care information were driven by a Spanish directive regarding the registry of specialized health care activities, which was prompted by the European initiative of defining minimum basic datasets (locally known as Conjunt Mínim Bàsic de Dades in Catalan) of hospital and outpatient activities as a foundation for the management of health care services [72]. Different claims registries were consecutively created to cover several stakeholders and care levels, including primary care, hospitalization, emergency room, emergency transportation, specialized outpatient care, intermediate care facilities, mental health hospitals, and outpatient mental health.

The claim registries differ in the type of information collected, although they share a similar structure that includes a unique identification number common for all registries of the Catalan Health Service. Similarly, the updating frequency depends on the purpose of each registry (Table 1).

The claim registries have an automated data validation system aimed at identifying inconsistencies between variables (eg, age-diagnosis correspondence and temporal sequences) and undergo external audits periodically to ensure provider payment accuracy

**Table 1:** Minimum basic datasets established by the Catalan Health Service.

Registry	Care level	Type of information	Variables, n	Year launched
Hospitalization	Specialized care	Admissions to hospitals, including stays and procedures	159	1992
Primary care	Primary care	Visits to primary care services (nurses or physicians)	82	2014
Emergency	Specialized care	Admissions to the emergency room	65	2012
Outpatient mental health	Specialized care	Outpatient visits to mental health professionals	75	2003
Mental health hospitals	Specialized care	Admissions to mental health hospitals	52	2003
Intermediate care	Intermediate care	Admissions to intermediate care and skilled nursing facilities	Palliative care: 31 and long-term care: 169	2003
Outpatients	Specialized care	Diagnoses and procedures of outpatient visits	72	2016

#### From Benchmarking to Population Health Assessment

The systematic collection of health information for health care claims, which included demographics, diagnoses, and use of health care resources, allowed for the development of registries aimed at public health surveillance. In 2011, the Catalan Health Surveillance System was created as an integrated platform periodically gathering data from claims registries and drug billing registries. This integration allowed for a comprehensive view of Catalan patients in a single database that included demographic characteristics (ie, age, sex, and socioeconomic status), clinical characteristics (ie, all chronic and acute diagnoses), and resource use (hospitalization, primary care visits, emergency department visits, skilled nursing facilities, palliative care, mental health services, pharmacy dispensation, outpatient visits to specialists, home hospitalizations, medical transportation [urgent and nonurgent], ambulatory rehabilitation, respiratory therapies, and dialysis).

The Catalan Health Surveillance System set up 2 important resources: the platform of modules for the follow-up of quality indicators (Mòduls per al Seguiment d’Indicadors de Qualitat in Catalan) and the Adjusted Morbidity Group (AMG) case-mix tool [73].

The modules for the follow-up of quality indicators are a business intelligence tool for the Catalan health care system, providing key performance and morbidity indicators at the hospital and emergency room levels. It offers data on demographics, health care interactions, and the prevalence of the top 20 chronic diseases available for the overall population and by health care area. In addition, it includes health care quality metrics such as in-hospital mortality, complications, readmissions, and preventable hospitalizations for each area.

More recently, the integration of indicators on morbidity and resource use has evolved toward public business intelligence for benchmarking, in which citizens and providers can review several key performance indicators among primary care teams and hospitals [74].

AMG is a case-mix tool that considers all possible chronic conditions (and recent acute diagnoses) among the diagnosis code groups of the International Classification of Diseases, 10th Revision, Clinical Modification [73,75]. On the basis of a model that accounts for differences in key health outcomes, the tool provides a morbidity index representing the clinical complexity of patients and allocates each patient to a morbidity group based on their complexity. The numerical index can be used to stratify the population into health risk groups. The AMG tool has shown high performance in predicting key health outcomes [7,73,76] and has been used as an adjusting variable to summarize the comorbidity burden in many research projects and internal benchmark analyses to account for the clinical complexity of individuals tended to by health care teams or providers being compared in the settings of research and resource allocation [77,78]. In the COVID-19 setting, a similar approach was used to develop a specific risk tool that predicted hospitalization or death beyond age [79].

## Discussion and Future

The transformation journey of the Catalan health care system over the past 25 years illustrates the efforts toward a paradigm of health care delivery also envisioned by other countries that is more integrated, efficient, and person centered. This transformation has occurred concomitantly with the adoption of HIT.

Revisiting the international examples mentioned in the Introduction section provides valuable context for the Catalan HIT journey. The ICSs of the National Health Service in the United Kingdom share similarities with Catalonia’s approach, particularly in their aim to better coordinate services across health and social care. However, while the UK ICS is a relatively recent initiative, Catalonia’s integrated care efforts, supported by its HIT infrastructure, have been evolving for >2 decades. This longer-term perspective offers insights into the challenges and benefits of this integration effort, which have been extensively reported elsewhere [78,80-84]. The Dutch vision on network medicine emphasizes connected care networks to address complex patient needs, an approach that aligns closely with Catalonia’s care process management platform. The implementation of this concept in Catalonia through tangible HIT solutions provides a practical example of how such a vision can be feasible. In comparison to the US patient-centered medical homes and neighborhoods, which focus on comprehensive, patient-focused care primarily in primary care settings, Catalonia’s approach extends this person-centric model across the entire health care continuum, facilitated by its integrated HIT systems. The Catalan experience, with integrated platforms such as the shared EHR, the care process management platform, or the federated platforms, demonstrates how a unified HIT infrastructure can support and enhance these person-centered care models on a system-wide scale despite the absence of a common health information system.

Catalonia’s early adoption of EHRs in the late 1990s and early 2000s placed the region at the forefront of the global EHR movement, aligning with trends observed in other European countries, although the pace of adoption varied significantly worldwide. A study by Jha et al [85] found wide variation in EHR adoption rates across 7 nations as of 2005. The Netherlands led with nearly 100% adoption in primary care, a trend that has continued and expanded to other countries [85]. As of 2016, countries such as Australia, the Netherlands, New Zealand, Israel, and Germany had achieved near-universal EHR adoption among physicians [85]. However, these rates of EHR adoption have not been mirrored in all high-income nations. Canada and the United States, for instance, had implementation rates as low as 25% by 2009 [86]. In response to this lag, the US federal government took decisive action through the Health Information Technology for Economic and Clinical Health Act, committing unprecedented resources of up to US $27 billion over 10 years to support the meaningful adoption and use of EMRs and EHRs through incentive payments [87]. This significant investment accelerated EHR adoption in the United States, highlighting the importance of governmental support and financial incentives in driving technological transformation in health care. In addition to governmental support, the progress of Catalonia in this area was facilitated by its unified health care model and single public insurer system, which provided a more coordinated environment for technology implementation compared to more fragmented health care systems. This structural advantage allowed Catalonia to achieve high adoption rates without the need for large-scale federal incentive programs such as those implemented in the United States.

Aside from the broad adoption of EHRs, Catalonia has made significant strides in patient-professional interaction through its “My Health” platform [46]. In this regard, it is important to also consider the advancements made by other European countries, particularly Denmark and Estonia, in this area. Denmark has been a pioneer in developing patient portals. The Danish eHealth portal [88], launched in 2003, provides citizens with comprehensive access to their health data and a range of eHealth services. According to Kierkegaard [89], by 2010, over 85% of the Danish population had used the portal. The portal allows patients to access their full medical records, book appointments, renew prescriptions, and communicate with health care providers. Denmark’s success in this area is attributed to its long-standing focus on eHealth and strong coordination between national and regional health authorities [89]. Estonia, known for its advanced digital society, has also made remarkable progress in eHealth. The Estonian eHealth system, launched in 2008, includes a patient portal that gives citizens access to their health records, e-prescriptions, and various e-services [90,91]. According to a case study conducted by Kivekäs et al [91] in 2017, a total of 47% of the Estonian population had accessed their health data through the portal. Estonia’s approach is notable for its integration of health data with other digital services through the country’s X-Road data exchange layer, providing a high level of interoperability and security. Both Denmark and Estonia’s experiences highlight the importance of strong national eHealth strategies, robust digital infrastructure, and a culture of digital innovation in driving the adoption and use of patient portals [92].

In our experience, in Catalonia, one of the critical drivers of this transformation has been the political commitment to a unified health care delivery model, facilitated by a single public insurer with direct economic leverage on service provider organizations. This foundational feature enabled the implementation of key infrastructures, such as the unique patient identifier, which became a cornerstone for subsequent integrations and innovations in the health care ecosystem. The lack of such a unique identifier, explicitly discouraged in some countries such as the United States, requires algorithmic approaches to information integration that can lead to errors and reduced quality of care [37]. On the other hand, unique identifiers have raised concerns regarding the privacy of patient information [37]. The historical willingness of Spanish governments to have a single and public health insurer that operates through a unique identifier has significantly facilitated the creation of HIT structures with a person-centered perspective and is a cornerstone of the Catalan eHealth platform.

The unique patient identifier was a key facilitator of the shared EHR launched in 2008, relatively early compared to many other regions. This initiative represents a significant step toward integrated care, aligning with broader international trends in health care digitalization and service integration. Denmark, often cited as a leader in HIT, launched its national health portal with shared EHR access in 2003. The Danish health data network allows health care providers to access patient data across different care settings, facilitating continuity of care and reducing duplication of services. By 2010, nearly all primary care physicians in Denmark were using EMRs and sharing data through the national network [89]. In the United Kingdom, the National Health Service has been working toward a more integrated approach to health records and care delivery. The National Health Service Long Term Plan, published in 2019, sets out a vision for ICSs across England. A key component of this strategy is the development of shared care records across health and social care providers within each ICS [17]. The aim is to enable seamless information sharing between different care settings, improving coordination and patient outcomes. While implementation is still ongoing, early adopter regions such as Greater Manchester have made significant progress in developing shared care records across their ICSs [17]. Similarly, in the United States, health information exchanges have been developed to facilitate the sharing of electronic health information across organizations within a region, community, or hospital system. For example, the Indiana Health Information Exchange, one of the oldest and largest in the United States, connects >100 hospitals and 14,000 practices, allowing providers to access patient data from multiple sources [93]. Australia has also made strides in this area with the My Health Record system, a national EHR system launched in 2012. By 2019, over 90% of Australians had a My Health Record, allowing them to share their health information with health care providers across the country [94].

The Catalan shared EHR system stands out for its comprehensive approach, integrating data from multiple providers across the region. The system allows health care professionals to access relevant patient information regardless of where it was generated within the Catalan health care system. This level of integration has been challenging to achieve in many other health care systems, particularly those with more fragmented provider landscapes. However, the implementation of shared EHR systems is not without challenges. Issues of data privacy, security, and interoperability remain significant concerns worldwide. A study by Fragidis and Chatzoglou [95] highlighted that, while many countries have made progress in implementing national EHR systems, barriers such as lack of standardization, resistance to change, and concerns about data protection continue to hinder full adoption and use. Despite these challenges, the trend toward shared EHRs continues to gain momentum worldwide, driven by the potential benefits of improved care coordination, reduced medical errors, and more efficient health care delivery.

Catalonia’s implementation of remote consultations in 2015 positioned the region at the forefront of telemedicine adoption, a trend that was dramatically accelerated by the COVID-19 pandemic. Initially, remote consultations were primarily used by younger patients for straightforward health concerns; however, the pandemic prompted a significant expansion of their use, becoming a crucial tool for maintaining health care services [48,59]. This shift mirrored global trends, with many countries rapidly adopting telemedicine to ensure continuity of care while minimizing viral transmission risks [96]. The response of the Catalan health system to this surge included the development of the “Latitud” model, an evidence-based framework for implementing remote consultations, addressing challenges such as technical issues and the need for physical examinations identified in other contexts [60,97]. The effectiveness of remote consultations in Catalonia has been promising, with retrospective reports indicating the resolution of 88% of cases, demonstrating a high potential for reducing health care center workloads [62]. However, challenges remain, including limited digital literacy among some patient groups and the need to integrate telemedicine effectively into professional workflows [59]. Looking forward, health care systems, including in Catalonia, are likely to adopt a hybrid model blending in-person and remote care. The Catalonia experience offers valuable insights for other health care systems, highlighting both the potential of remote consultations to improve health care accessibility and efficiency and the importance of evidence-based implementation strategies to ensure quality of care in this new paradigm.

The limitations of the Catalan digital health infrastructure became increasingly apparent as the demand for more integrated and patient-centered services grew [5,84,98,99]. The fragmented nature of health care data, confined within proprietary systems and inaccessible across different health care settings, led to inefficiencies, effort duplication, increased costs, and barriers to the scaling up of innovation. Moreover, the rising expectations of patients, who increasingly sought more involvement in their health care decisions and access to their health information, underscored the need for a more open and connected approach [100]. Finally, it is worth mentioning that, despite the large amount of data routinely collected and centralized by the Catalan Health Service, many decisions regarding planning and resource allocation are not yet data driven and most health indicators of the Catalan Department of Health still rely on population surveys, as they did years before [30]. This scenario suggests an underuse of the data that goes beyond technological barriers.

**Figure 8:**
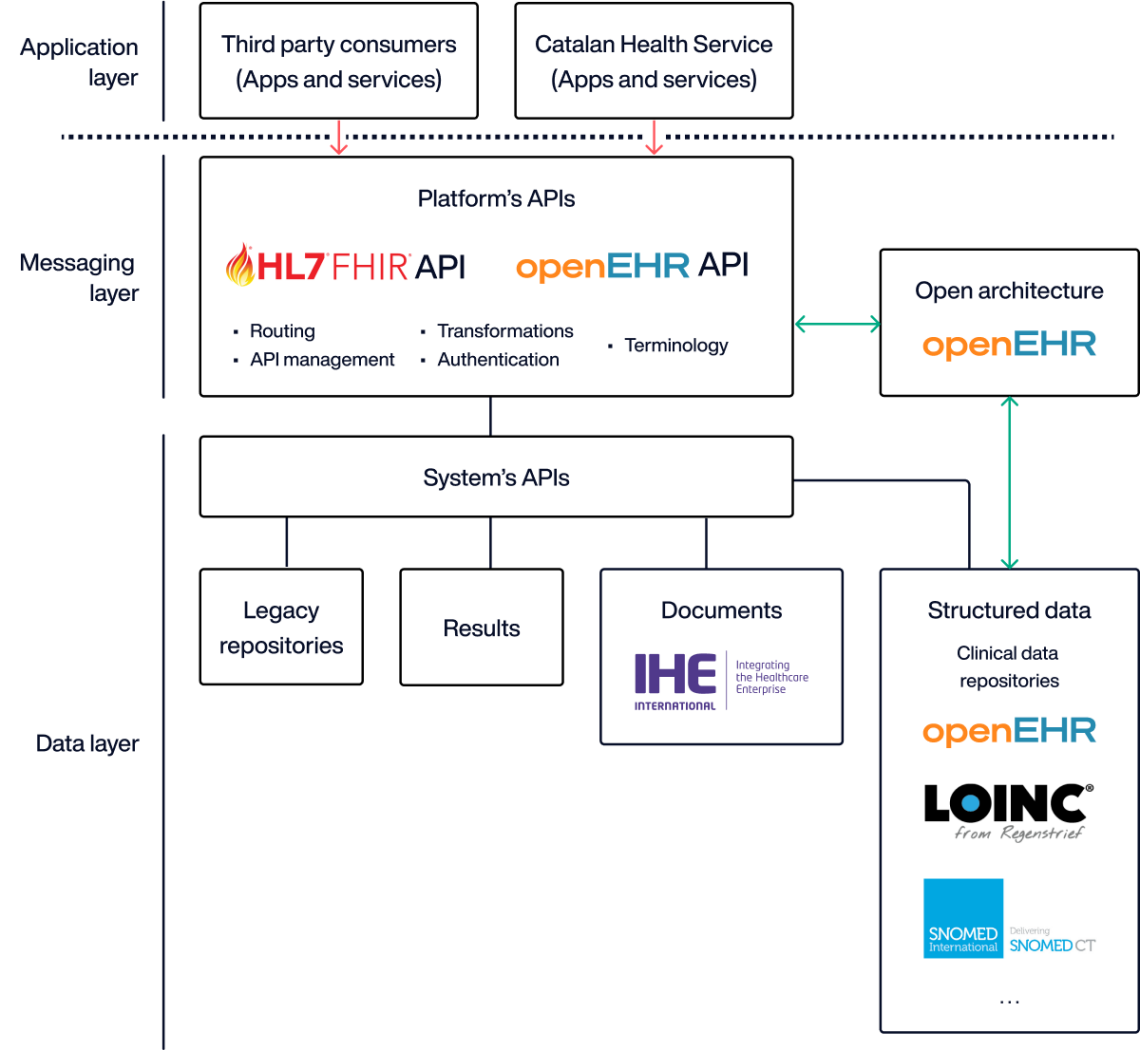
Architecture of the envisioned open platform of the Catalan Health Service (adapted from Defining an Open Platform—Apperta Foundation). API: application programming interface.

Recognizing these challenges, Catalonia embarked on a journey toward the open platform paradigm in health as defined in the digital health strategy for Catalonia [57]. The idea of the open platform is a forward-thinking approach first defined by the Apperta Foundation, which emphasizes the importance of interoperability, open standards, patient-centered care, and the strategic use of digital technologies to enhance health outcomes [101]. It entails a shift away from proprietary, siloed systems toward a more integrated, flexible, and user-friendly digital health ecosystem. This paradigm advocates for the idea that health care should be accessible, transparent, and seamlessly connected, enabling the free flow of health data to support patient care, research, and innovation. The underpinning concept is that information systems for patient data collection and clinical information share the need for an information architecture (ie, International Organization for Standardization 18308:2011) based on standardized or logical information models [102]. This element is key for a satisfying experience for users—both professionals and citizens—in all their interactions with the health system tools, facilitating updates and innovations in EHR-related applications used by health care providers, and centralizing patients' personal health data. The technological progress makes it essential to push the current deployment of HIT from interoperability to shared and common vocabularies and ontologies, achieving full data liquidity between organizations and systems through the meaningful use of different standards [103] (Figure 8).

Looking ahead into the future, Catalonia will continue to evolve its HIT infrastructure on the open platform paradigm. The focus will be on further enhancing integration, leveraging advanced technologies, and improving patient engagement. A key priority is the seamless integration of health and social care data, which would support a more holistic approach to patient care and enable better management of complex, chronic conditions. Concurrently, there is significant potential in harnessing artificial intelligence to improve clinical decision support systems and refine population health management strategies. These advanced analytics could provide invaluable insights for preventive care and resource allocation. Patient empowerment remains a crucial area for development, with opportunities to expand and improve digital tools that facilitate active participation in health care decisions, including better telemedicine platforms and more intuitive interfaces for accessing health care services. In addition, addressing any remaining interoperability challenges is vital to ensure seamless data flow across all health care settings, from primary care to specialized hospital services. This would not only improve care coordination but also support more comprehensive research and public health initiatives.

As final remarks, for countries embarking on their HIT journey, the Catalan experience offers several key considerations:

- Political and structural foundations—a unified health care model and strong political commitment are crucial for successful implementation.
- Phased approach—start with foundational elements such as unique patient identifiers and basic EHR systems before moving to more advanced integrations.
- User-centric design—prioritize the needs of both health care providers and patients in system design to ensure adoption and effectiveness.
- Data governance—establish robust data privacy and security measures from the outset to build trust and ensure compliance with regulations.
- Interoperability—adopt international standards for data persistence and exchange to facilitate future integrations and avoid vendor lock-in.
- Change management—invest in training and support for health care providers to ensure smooth adoption of new technologies.
- Continuous evaluation—regularly assess the impact of HIT implementations on health care quality, efficiency, and patient outcomes to guide future developments.

## Conclusions

The evolution of HIT in Catalonia over the past quarter of a century provides valuable insights and lessons for other regions aiming to navigate the complex landscape of digital health care transformation. However, the effectiveness of such strategies is contingent on a robust primary care network and a unified health care model. Hence, the scope of our analysis and applicability of most of the solutions described in this paper are particularly suited to countries with universal health care coverage, a unique patient identification number, and a strong network of primary care centers.

The future of health care digitalization in Catalonia and potentially elsewhere hinges on overcoming legacy data fragmentation and moving toward a genuinely integrated health and social information system that streamlines the strategic use of health data. This transition is not just about adopting new technologies but also about a paradigm shift in health care delivery, moving from referrals and transferability of responsibility toward a collaboration emphasizing a holistic approach to patient care for improved health outcomes.

## Appendix

Multimedia Appendix 1 Additional information on the drug prescription system, recommendations for the use of remote consultations, and supplementary tables.

## Abbreviations

AMG: Adjusted Morbidity Group
EHR: electronic health record
EMR: electronic medical record
ePROM: electronic patient-reported outcome measure
FHIR: Fast Healthcare Interoperability Resources
GP: general practitioner
HIT: health information technology
HL7-CDA: Health Level 7 Clinical Document Architecture
ICS: integrated care system
LOINC: Local Observation Identifiers Names and Codes
NHS: National Health System
PROM: patient-reported outcome measure
SIMDCAT: Digital Medical Imaging System of Catalonia
SNOMED-CT: Systemized Nomenclature of Medicine–Clinical Terms
