# Supplementary material for: A 25-Year Retrospective of Health IT Infrastructure Building: The Example of the Catalonia Region
Source: J Med Internet Res. 2024 Nov 18;26:e58933. doi: 10.2196/58933 (PMC11612585; doi:10.2196/58933)
Supplement: Multimedia Appendix 1 [file jmir_v26i1e58933_app1.pdf]

A 25-YEAR RETROSPECTIVE OF HEALTH IT INFRASTRUCTURE BUILDING: THE EXAMPLE OF THE CATALONIA REGION

Supplementary material

Contents

Supplementary descriptions 2
Drug prescription system 2
Recommendations for the use of Remote consultations 3
Recommendations for professionals 3
Recommendations for healthcare organizations........ 10
Supplementary Tables........ 14
Table S1. Characteristics of the information stored in the shared electronic health record of Catalonia. 14
Table S2. Current storage of the SIMDCAT federated platform according to diagnostic imaging techniques (from inception and until 31/12/2023) 18
Table S3. ePROMs implemented within the Catalan Healthcare system. 19
Supplementary References........ 22

Supplementary descriptions

Drug prescription system

Outpatient drugs are prescribed by physicians (either GPs or specialists) providing care through public coverage and dispensed at private community pharmacies based on a co-payment system. Community pharmacies in Spain are regulated in number and location (i.e., fixed ratio per inhabitant and minimum distance between them) and have an agreement with the Healthcare Department for reimbursement. Drugs are co-payed based on socioeconomic status, with contribution by citizens ranging from 0% to 60%, and an approximate average of 11%. Drug prices are established nation-wide by the Spanish Ministry of Health, regardless of the co-payment or reimbursement status.

The categories of pharmaceutical co-payment are defined based on four levels of annual income as follows: recipients of social services (i.e., individuals perceiving a minimum integration income, unemployment allowance, unemployment benefit, or not qualifying for either of the previous, those on leave for work-related accident or professional disease), low socioeconomic status (i.e., individuals with annual incomes below € 18,000), middle socioeconomic status (i.e., individuals with annual incomes between € 18,000 and € 100,000), and high socioeconomic status (i.e., individuals with annual income higher than € 100,000). In addition, in 2021 additional categories for pharmaceutical co-payment exemption have been implemented (those with minimum vital subsidy, those with subsidies for a dependent child or minor in a permanent family foster regime or guardianship for adoption purposes; minors with a recognized disability of 33% or higher, and individuals perceiving pensions with an annual income lower than 11,200 €). The criteria and categories of pharmaceutical co-payment are currently registered in a central database of users (*Registre Central d'Assegurats* or RCA). The co-payment status is applied for the entire year according to the status by January 1st of the year in RCA. Individuals younger than 18 years are assigned according to their parents' status.

Recommendations for professionals

Recommendations for the use of non-face-to-face care channels

Professionals in specialized care centres, intermediate and long-term care centres, and mental health and addiction centres

Recommendations for the use of **non-face-to-face care channels** for health professionals

In order to face a situation of growing demand, with scenarios of restricted mobility for the citizens, changes in habits and trends in consumption of digital services, **new channels of non-face-to-face care are being incorporated.** This sheet of recommendations is intended to support **outpatient professionals in hospitals, social health centers and mental health centers** to identify situations where it is possible to use these channels, and inform them of the aspects to be taken into account when using the available channels: telephone, eConsultation (secure messaging) and videoConsultation.

Progressively, these non-face-to-face care **channels** will be integrated within **"La Meva Salut" the digital health portal**, so that the citizens can access from this portal. This enables to have a unique platform of relationship between the health system and the citizens that gathers **channels** and services, and guarantees the security and consent of the information.

Citizens must be told to register in **the digital health portal "La Meva Salut" and informed about the services it offers**: agenda, previous appointment, access to the eConsultation and soon to the videoConsultation, consultation and downloading diagnostic test results, reports, medication plan, etc.

La meva /Salut

To register in "La Meva Salut", you can do it through the following form:

- **lamevasalut.gencat.cat/alta**
- or calling to **900 053 723** (Monday to Friday from 8.00am to 8.00pm)

Moments in the care process when I can provide non-face-to-face care

Diagnostic Process · **Surgical Procedure** · **Follow-up, treatment and monitoring** (ambulatory or at home)

Examples of types of visits through non-face-to-face care channels

eConsultation — asynchronous

- Non-critical **treatment** adjustments.
- Information on **test preparation and analysis**.
- Test and analytical results with non-critical **results**.
- Resolution of **specific doubts** about treatments.
- **Follow-up** visits.

Telephone call — synchronous

- **Preparation** of the **initial visit.**
- Preparation of the **surgical process** (previous information).
- **Non-critical treatment** adjustments.
- Test and analytical results with non-critical **results**.
- **Follow-up visits**.

videoConsultation

- **Health education.**
- **Preparation of face-to-face visits.**
- Non-critical **treatment** adjustments.
- **Previous visits for minor surgeries** (e.g. Anesthesiology).
- **Follow-up of care** that does not involve face-to-face interaction.
- **Follow-up** visits.
- Resolution of **doubts about therapies and treatments.**

Criteria to be considered for non-face-to-face visits

Care Criteria

- **Severity and complexity** of the citizen and its situation.
- Need to perform **physical exploration, visualize the citizen in person** and / or perform techniques that involve face-to-face care.
- **Ability to solve the visit** (can I fulfill the objective of the visit with this modality?).
- **Nature of the information** to be communicated.
- **Privacy** assurance of the visit.
- Guarantee of the **protection and security** of the data generated during the visit.
- **Normative / legal** aspects.

Criteria related to the citizen

- **Preferred** mode of care.
- **Ability to understand** the information.
- **Mobility** of the citizen.
- Possibility of **involvement of the caregiver**.
- Consider if the visit is with a **known person or not**.

Summary | Channels | Legal aspects | Good Practices

2020. © Generalitat de Catalunya. Servei Català de la Salut (2.7.2020)

Generalitat de Catalunya

Funded by the European Union though the Structural Reform Support Programme

/Salut

Channels and requirements

eConsultation

- The **eConsultation** service enables the delivery of messages through a secure channel to citizens.
- It also **enables the attachment** of images, reports and other files.
- It is an asynchronous channel that offers **flexibility** in response.
- It is **initiated by the specialized, intermediate and long-term care professional** but, while the episode lasts, the citizen can also initiate conversations.

Telephone Call

- The **phone service** is a synchronous communication channel that only requires network coverage and a phone to make the visit.
- It is an adequate channel in visits where visual contact is not necessary.
- **It is initiated by the professional.**
- If, as a result of the **telephone care**, it is necessary to send to the citizen the results of diagnostic tests, reports or a new medication plan, this can be done through **"La Meva Salut"**

videoConsultation

- The **VideoConsultation** service allows to make audio and video calls using a computer, tablet or smartphone.
- This channel is suitable when attending in person is not required and visual contact allows a higher degree of resolution of the visit.
- **It is initiated by the professional.**
- If, as a result of the **videoConsultation**, the citizen needs to receive the results of diagnostic tests, reports or a new medication plan, this can be done through **"La Meva Salut"**.

Legal aspects

Before starting

- Comply with data protection and privacy requirements before carrying out a visit (already integrated in the videoConsultation and eConsultation tools and, depending on the healthcare provider, in the telephone calls).
- Ensure that the citizens have given their consent, at least verbally, and that it is recorded in their medical records.
- Ensure that the citizen and the professional can carry out the consultation without violating their privacy.

During the non-face-to-face care

- Respect the privacy of the citizens in accordance with the legislation in force to carry out the teleconsultations.

The right to information

- Information about the privacy aspects can be consulted in the privacy policy and the conditions of use of the non-Face-to-Face Care.
- The citizen has the right to be informed about the use and treatment of the data generated during the care received.

Conduct and good practices

Main behavioral aspects and good practices to be considered

Before starting

- **Have time scheduled** in the agenda to make the visit.
- Review the **reason for the consultation**, the citizen's background and the ability to solve the consultation with the chosen channel.
- Ensure **availability and capacity** of the citizen to use the channel.
- Ensure coverage and / or access to the network / Internet.
- Learn the code of conduct to perform the appropriate attention to the situation.

During the visit

- Identify **yourself and the citizen**.
- Ensure the absence of connectivity problems with the citizen during the visit.
- **Communicate in a clear, understandable and adapted way** to the profile of the citizen.
- Verify that the citizen has understood the messages and understands the next steps.
- Be **careful with the assessment of the case**, the lack of direct contact may affect the capacity of evaluation.

After the visit

- Know the administrative procedures and the subsequent steps after the visit.
- **Incorporate the information** (clinical interpretation) to the medical record.

When does teleconsultation MAY NOT be recommended?

- First visits without prior case information
- Communication of sensitive information
- Visits where physical examination is required
- Follow-up visits for serious or highly complex cases
- In case of regulatory or legal contraindications
- If the fluency of the consultation cannot be guaranteed

La meva /Salut

Summary | Channels | Legal aspects | Good Practices

2020. © Generalitat de Catalunya. Servei Català de la Salut (2.7.2020)

Generalitat de Catalunya

Funded by the European Union though the Structural Reform Support Programme

/Salut

Requirements of **non-face-to-face care channels**

VideoConsultation Synchronous communication with audio and video

The **videoConsultation** service allows to make calls with audio and video.

The access by the citizens is done **through PC, smart phone or tablet.**

The **videoConsultation** can only be initiated by the healthcare professional

1 / Scheduling of visits

- The citizen must be informed of the details of the consultation: the terms and conditions of this channel, how to connect and what will be done in the event of not being able to establish contact.
- The health center must define who is in charge of scheduling the visit and notify the citizen.
- From the clinical station, a new videoConsultation appointment must be created, which will notify by e-mail or SMS to the citizen about the day, time and service of the appointment.
- This visit must be associated with the professional's agenda.
- The citizen will be informed that the access to the videoConsultation will be available soon through **"La Meva Salut"** digital health portal.

2 / Start of the visit

- The citizen will be informed that, if as a result of the videoConsultation it is necessary to send him/her the results of diagnostic tests, reports or a new medication plan, he/she will find them in **"La Meva Salut".**
- The professional must adapt the space where the visit will take place and adjust the camera to the height of the eyes.
- On the **day and time** indicated, the professional must access the **virtual waiting room**, through the clinical station.
- Once the citizen is also connected, the professional will receive a notice that the consultation can begin.

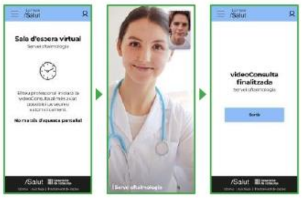

3 / At the end

- Before ending, it must be ensured that the citizen has understood the information given and is clear about what the next steps are.

The clinical interpretation of the visit must be incorporated into the medical record, as is done in a face-to-face visit.

When IS it appropriate?

/ When the videoConsultation enables to solve the reason of the visit.
/ If the presence of the citizen is not required.
/ When the visual contact helps to a better communication.
/ When the availability of the video allows to give an answer to the reason of the consultation.
/ If the severity and complexity of the citizen allows it.
/ When the professional is responsible for the case, or he/she is aware of it.
/ If it provides information prior to a face-to-face visit.
/ When the privacy of the visit can be ensured (professional and citizen).

When MAY NOT be recommended?

/ For first visits without previous information of the case.
/ To communicate sensitive information to the citizen.
/ For visits in which the citizen must be physically examined.
/ In follow-up visits to serious cases.
/ When there is a legal or regulatory contraindication (minors under 16 years old, people under guardianship, dependents).
/ When the availability of the channel by the citizen or his ability to use it cannot be ensured.
/ When Internet access that allows the professional to carry out the video consultation fluently cannot be ensured.

Citizens must be told to register in **the digital health portal "La Meva Salut" and informed about the services it offers**: agenda, previous appointment, access to the eConsultation and soon to the videoConsultation, consultation and downloading diagnostic test results, reports, medication plan, etc.

La meva /Salut

To register in "La Meva Salut", you can do it through the following form:

- **lamevasalut.gencat.cat/alta**
- or calling to **900 053 723** (Monday to Friday from 8.00am to 8.00pm)

Summary | Channels | Legal aspects | Good Practices

2020. © Generalitat de Catalunya. Servei Català de la Salut (2.7.2020)

Generalitat de Catalunya

Funded by the European Union though the Structural Reform Support Programme

/Salut

Legal and data protection aspects for telephone call, eConsultation and videoConsultation

The use of the **non-face-to-face care** channels means the transmission of personal data, especially health data, so it is necessary to ensure compliance with the regulations on data protection and privacy.

1 The right to information — How can I inform properly?

There must always be the will and **consent (at least verbal) of the citizen,** and this must be recorded in the medical record.

The citizen must receive information about:

- The provision of care through these channels and accept it through an agreement with the professional.
- The functioning of telematic channels.
- The treatment of their personal data.

This information can be consulted in the privacy policy and the terms of use of the Non-face-to-face care channels (eConsultation and videoConsultation).

/ Code of Ethics
/ Clinical standards
/ Data protection

Good practices

/ Be informed: be clear about the ethical and deontological code defined by your professional association.

/ Inform: provide citizens with clear and understandable information.

2 Before starting — How to ensure compliance?

The use of telematic channels or other non-face-to-face communication systems will be carried out according to the ethical code.

/ Privacy policy and terms of use

- Make sure that you have already agreed with the citizen to carry out this non-face-to-face activity.
- The privacy policy and the terms of use are available in the link of the corresponding teleconsultation channel.

/ Confidentiality during the visit
/ Identification of the participants

- The confidentiality and privacy of the citizen must be ensured. It is a priority to protect privacy in the professional-citizen relationship.
- The correct identification of the interlocutors (professional and citizen) is essential.

3 During the non-face-to-face consultation

Maintaining an attitude of respect for the citizen's privacy is essential.

- Make sure you have access to the appropriate software to perform the visit and record the health data.
- The treatment of personal data follows the same regulation as in the face-to-face visits.
- Register to the medical record the data and the activity that is essential.

Good practices

/ Do not use personal devices or addresses.

/ Use only those channels given by the provider centre.

Current regulations

General Data Protection Regulation 2016/679.

Organic Law 3/2018, on the protection of personal data and guarantee of digital rights.

Law 41/2002, on patient autonomy and rights and obligations regarding clinical information and documentation.

Law 21/2000, on the rights of information concerning the health and autonomy of the patient, and clinical documentation.

Portfolio of rights and duties of citizens in relation to health and health care.

2020. © Generalitat de Catalunya. Servei Català de la Salut (2.7.2020)

Generalitat de Catalunya

Funded by the European Union though the Structural Reform Support Programme

/Salut

Good practices manual

Recommendations of good practices when making non-face-to-face visits with the available channels in the Catalan health system (phone call, eConsultation and videoConsultation).

Cross-cutting elements to the various non-face-to-face channels

- Make the previous evaluation of the case and background.
- Access the medical record and other documentation required to make the visit.
- Guarantee the necessary conditions to make the visit properly and without interruptions.
- Ensure the confidentiality and privacy of the visit (for both the professional and citizen).
- Reserve time in the agenda for the visit and do not do other activities simultaneously.
- Check the correct functioning of the non-face-to-face care channels.
- Reserve a few minutes to involve the citizen when the visit is with the caregiver or guardian.
- A cautious attitude must be maintained with the evaluation of the non-face-to-face visit in order to guarantee the correct evaluation of the case.

Specific elements to carry out the visit with the different channels

	eConsultation	Phone call and VideoConsultation
**1 Before the visitit**	• Please note that the citizen, once the visit is scheduled in the agenda, will receive a message indicating that a response will be received within 48 hours.<br>• The eConsultation can be initiated by both the citizen and the professional.	• Check the contact of the citizen (name, phone number, Individual health card number).<br>• Consider the presence of an accompanying person to the visit and confirm their contact details if is in a different location from the attended citizen.<br>• Check the proper functioning of the audio and / or video and check that the device has sufficient battery.<br>• In the case of a video call, take into account: sufficient light in the consultation room, professional atmosphere, professional clothing and positioning of the camera at the eye level of the professional.
**2 During the visit**	• Give your greetings at the beginning and end of the visit.<br>• Identify yourself.<br>• Use a close, clear and easy to understand wording, adapted to the profile of the citizen.<br>• Avoid writing the message in capital letters.<br>• Structure the message in paragraphs.<br>• Write clearly the therapeutic guidelines and recommendations regarding treatment.<br>• Check the message before sending it.<br>• Make sure that the message has been sent.	• Identify yourself and the citizen.<br>• Explain how the visit will be approached.<br>• Inform the citizen that the session is private and confidential.<br>• Confirm that the citizen has privacy to carry out the visit.<br>• Confirm that the citizen has time, battery, coverage and connectivity.<br>• Conduct the clinical interview with: active listening, empathy and giving clear, easy-to-understand messages adapted to the profile of the citizen.<br>• Summarize the visit and check that the citizen has understood the information given and the next steps (if necessary).

Citizens must be told to register in **the digital health portal "La Meva Salut" and informed about the services it offers**: agenda, previous appointment, access to the eConsultation and soon to the videoConsultation, consultation and downloading diagnostic test results, reports, medication plan, etc.

La meva /Salut

To register in "La Meva Salut", you can do it through the following form:

- **lamevasalut.gencat.cat/alta**
- or calling to **900 053 723** (Monday to Friday from 8.00am to 8.00pm)

3 After the visit

- Send the agreed documentation / information.
- Incorporate the clinical interpretation of the visit into the medical record, just as it is done in a face-to-face visit.
- Plan the corresponding follow-up tasks.

2020. © Generalitat de Catalunya. Servei Català de la Salut (2.7.2020)

Summary | Channels | Legal aspects | Good Practices

Generalitat de Catalunya

Funded by the European Union though the Structural Reform Support Programme

/Salut

Requirements of **non-face-to-face care channels**

Telephone Call Synchronous communication with audio

1 / Scheduling of visits

- It is necessary to define in the scope of the health center who is the one in charge of scheduling the visit and notifying it to the citizen.
- The citizen must be informed of the details of the visit: the terms and conditions of this channel, how they will be contacted and what will be done in case they cannot establish contact.
- The visit must be linked to the professional's agenda.

2 / During the consultation

- It should begin with an initial dialogue to identify the professional and the citizen.
- The objective of the visit should be explained.
- It is necessary to explain what to do in case the connection is lost *.
- Confidence must be built with the interlocutor.
- Do not interrupt the citizen when he/she is speaking, listen to him/her empathetically.
- They should be asked to listen carefully and, in case of doubt, to ask them.
- The citizen must be informed that, in case it is necessary to send them the results of diagnostic tests, reports or a new medication plan, they will find them in "La Meva Salut".

3 / At the end

- Before ending, it must be ensured that the citizen has understood the information provided and is aware of what the next steps are.
- The clinical interpretation of the visit must be incorporated into the medical record, just as it is done in a face-to-face visit.

When IS it appropriate?

/ When the communication through telephone allows to solve the reason of the visit.
/ If the attendance in person of the citizen is not required.
/ When the severity and complexity of the situation and / or pathology of the citizen allows it.
/ If the professional is responsible for the case, or knows the case.
/ When information is provided prior to an on-site visit.
/ When the privacy of the visit can be ensured (professional and citizen).

When MAY NOT be recommended?

/ For first visits without previous information of the case.
/ To communicate sensitive information to the citizen.
/ For visits in which the citizen must be physically examined.
/ In follow-up visits to serious cases.
/ When there is a legal or normative contraindication (under 16 years old, people under guardianship, dependent people).
/ If it cannot be ensured that the channel allows the professional to make the consultation fluently.

Citizens must be told to register in **the digital health portal "La Meva Salut" and informed about the services it offers**: agenda, previous appointment, access to the eConsultation and soon to the videoConsultation, consultation and downloading diagnostic test results, reports, medication plan, etc.

La meva /Salut

To register in "La Meva Salut", you can do it through the following form:

- **lamevasalut.gencat.cat/alta**
- or calling to **900 053 723** (Monday to Friday from 8.00am to 8.00pm)

2020. © Generalitat de Catalunya. Servei Català de la Salut (2.7.2020)

Generalitat de Catalunya

Funded by the European Union though the Structural Reform Support Programme

/Salut

Requirements of **non-face-to-face care channels**

eConsultation

Secure Asynchronous Messaging

The **eConsultation** service allows the delivery of written messages through a secure channel.
The **eConsultation** service enables the attachment of images, reports and other files.

1 / Start

- The citizen must be informed of the eConsultation channel before using it for the first time.
- The **eConsultation** is initiated by the professional and then can be initiated by both the citizen and the professional.
- The **eConsultation** allows the citizen to inform about the reason of the consultation.

/ Scheduled follow-up
/ Clinical deterioration
/ Consultation on diagnostic tests
/ Medication consultation
/ Possible adverse effects
/ Request for documentation
/ Clinical incidence
/ Scheduling of visits
/ Others

2 / Answer

- It is necessary to answer in a clear and simple way, and to indicate the steps to follow.
- If needed, the medication plan must be updated or other actions related to the reason for the consultation must be carried out.
- You must indicate the need or not to carry out successive activities.
- The citizen must be informed that he or she can find the results of tests, reports, diagnoses and the medication plan at "La Meva Salut".

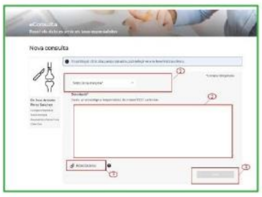

3 / End

- It is necessary to ensure that the answer is clear and understandable and that the citizen is informed on how to proceed afterwards.
- The clinical interpretation of the visit must be included in the citizen's medical record, just as it is in a face-to-face visit.

When IS it appropriate?

/ When attendance in person is not required.
/ If the consultation can be resolved by text message.
/ To solve specific doubts of citizens.
/ To provide indications prior to tests, analyses or face-to-face visits.
/ To make small adjustments in the treatment.
/ To communicate the publication of results to "La Meva Salut".
/ For any procedure request (clinical report, medication plan update, publication of discharges due to temporary disability to "La Meva Salut", test management, etc.).
/ To request tests or analysis by the professional after a follow-up eConsultation.
/ To resolve incidents or consultations resulting from a test or analysis.
/ To explain the results of tests..

When MAY NOT be recommended?

/ For initial visits without prior case information
/ To communicate sensitive information to the citizen
/ On visits where the citizen must be physically examined
/ In follow-up visits for serious cases
/ When there is a normative or legal contraindication (under 16 years old, people under guardianship, dependants)
/ When the citizen's ability to use the channel cannot be ensured
/ When Internet access cannot be ensured

Citizens must be told to register in **the digital health portal "La Meva Salut" and informed about the services it offers**: agenda, previous appointment, access to the eConsultation and soon to the videoConsultation, consultation and downloading diagnostic test results, reports, medication plan, etc.

La meva /Salut

To register in "La Meva Salut", you can do it through the following form:

▶ **lamevasalut.gencat.cat/alta** ▶ or calling to **900 053 723** (Monday to Friday from 8.00am to 8.00pm)

2020. © Generalitat de Catalunya. Servei Català de la Salut (2.7.2020)

Generalitat de Catalunya

Funded by the European Union though the Structural Reform Support Programme

/Salut

Recommendations for healthcare organizations

Recommendations for the use of non-face-to-face care channels

Directorate of specialized care centres, intermediate and long-term care centres, and mental health and addiction centres

Organizational recommendations for the use of the phone call, the eConsultation and the videoConsultation

The provision of services by means of **Non-face-to-face care** requires, in the organizational environment, to have assistance and management processes that include this modality of care and to guarantee the necessary competences on the part of the professionals. **This card is addressed to the providers centers**, to guide on the necessary activities for the implementation of non-face-to-face care channels.

Care processes with non-face-to-face care

Care Model

It is recommended to place non-face-to-face care in a model that enables a combination of face-to-face and non-face-to-face care. We must take into account aspects related to the health situation of the citizen, its conditions (socioeconomic environment, digital skills, etc.), and the resources available.

/ The plan of care must be agreed with the citizen and his or her consent must be obtained, even if it is verbal

/ The mechanism for recording the activity must be defined at the centre

Citizens who are candidates to receive non face-to-face care: example of an inclusion circuit

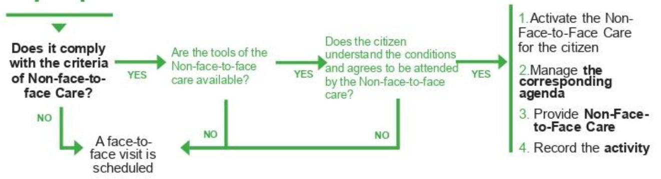

Aspects to consider:

- / Types of visits and selection criteria
- / Protocols of action and roles of professionals
- / Criteria for inclusion and exclusion of the citizen
- / Integration of Non-face-to-face Care in the professionals agenda

Key aspects

/ Protocols

It is important to establish clear and consensual protocols with the care management and IT departments for the use and integration of channels in daily practice.

It is necessary to specify which are the procedures to be carried out in case the connection is lost (phone call / videoConsultation).

/ Change management

It is essential to consider the perspective of the professional and the citizens in the change management towards a model that combines face-to-face and non face-to-face care. It is recommended to identify team members who support the change.

/ Communication and training

It is necessary to ensure proper training and information to support the change management in an appropriate way in the use of the various channels of non-face-to-face care.

Organization and competences

The use of Non-face-to-face care channels implies ensuring a minimum of organization requirements, roles and competences of the professionals.

Organizational aspects

- / Role of the IT department
- / Workflows and roles of professionals
- / Record of the activity
- / Administrative support processes for non-face-to-face care
- / Agenda management
- / Incident Management

Competence aspects

- / Digital skills
- / Training on non-face-to-face care channels
- / Citizenship training
- / Training for professionals
- / Codes of conduct
- / Technical support

Good practices

/ Training: it is necessary to ensure training in the use of the channels for non-face-to-face care of professionals

/ Support: administrative mechanisms must be defined to plan and confirm non-face-to-face activity

2020. © Generalitat de Catalunya. Servei Català de la Salut (2.7.2020)

Organization | Legal aspects | Checklists | Good Practices

Generalitat de Catalunya

Funded by the European Union though the Structural Reform Support Programme

/Salut

Legal and data protection aspects for phone call, eConsultation and videoConsultation

The use of the **non-face-to-face care** channels means the transmission of personal data, especially health data, so it is necessary to ensure compliance with the regulations on data protection and privacy.

1 The right to information — How can I inform properly?

There must always be the will and **consent (at least verbal) of the citizen,** and this must be recorded in the medical record.

The citizen must receive information about:

- The provision of care through these channels and accept it through an agreement with the professional.
- The functioning of telematic channels.
- The treatment of their personal data.

This information can be consulted in the privacy policy and the conditions of use of the Non-face-to-face Care channels (eConsultation and videoConsultation).

/ Code of Ethics
/ Clinical standards
/ Data protection

Good practices

/ Be informed: be clear about the ethical and deontological code defined by your professional association.

/ Inform: provide citizens with clear and understandable information.

2 Before starting — How to ensure compliance?

The use of telematic channels or other non-face-to-face communication systems will be carried out according to the ethical code.

/ Privacy policy and terms of use

- Make sure that you have already agreed with the citizen to carry out this non-face-to-face activity.
- The privacy policy and the conditions of use are available in the link of the corresponding teleconsultation channel.

/ Confidentiality during the visit / Identification of the participants

- The confidentiality and privacy of the citizen must be ensured. It is a priority to protect privacy in the professional-citizen relationship.
- The correct identification of the interlocutors (professional and citizen) is essential.

Current Regulations

General Data Protection Regulation 2016/679.

Organic Law 3/2018, on the protection of personal data and guarantee of digital rights.

Law 41/2002, on patient autonomy and rights and obligations regarding clinical information and documentation.

Law 21/2000, on the rights of information concerning the health and autonomy of the patient, and clinical documentation.

Portfolio of rights and duties of citizens in relation to health and health care.

3 During the non-face-to-face consultation

Maintaining an attitude of respect for the citizen's privacy is essential.

- Make sure you have access to the appropriate software to perform the visit and record the health data.
- The treatment of personal data follows the same regulation as in the face-to-face visits.
- Register to the medical record, the data and the activity that is essential.

Good pactices

/ Do not use personal devices or addresses.

/ Use only those channels given by the provider center.

2020. © Generalitat de Catalunya. Servei Català de la Salut (2.7.2020)

Generalitat de Catalunya

Funded by the European Union though the Structural Reform Support Programme

/Salut

Good practices manual

Recommendations of good practices when making non-face-to-face visits with the available channels in the Catalan health system (phone call, eConsultation and videoConsultation).

Cross-cutting elements to the various non-face-to-face channels

- ✔ Make the previous evaluation of the case and background.
- ✔ Access the medical record and other documentation required to make the visit.
- ✔ Guarantee the necessary conditions to make the visit properly and without interruptions.
- ✔ Ensure the confidentiality and privacy of the visit (for professional and citizen).
- ✔ Reserve time in the agenda for the visit and do not do other activities simultaneously.
- ✔ Check the correct functioning of the non-face-to-face care channels.
- ✔ Reserve a few minutes to involve the citizen when the visit is with the caregiver or guardian.
- ✔ A cautious attitude must be maintained with the evaluation of the non-face-to-face visit in order to guarantee the correct evaluation of the case.

Specific elements to carry out the visit with the different channels

1 Before the visitit

eConsultation

- Please note that the citizen, once the visit is scheduled in the agenda, will receive a message indicating that a response will be received within 48 hours.
- The eConsultation can be initiated by both the citizen and the professional.

Phone call and VideoConsultation

- Check the contact of the citizen (name, phone number, Individual health card number).
- Consider the presence of an accompanying person to the visit and confirm their contact details if they are in a different location from the attended citizen.
- Check the proper functioning of the audio and / or video and check that the device has sufficient battery.
- In the case of a video call, take into account: sufficient light in the consultation room, professional atmosphere, professional clothing and positioning of the camera at the eye level of the professional.

2 During the visit

eConsultation

- Give your greetings at the beginning and end of the visit.
- Identify yourself.
- Use a close, clear and easy to understand wording, adapted to the profile of the citizen.
- Avoid writing the message in capital letters.
- Structure the message in paragraphs.
- Write clearly the therapeutic guidelines and recommendations regarding treatment.
- Check the message before sending it.
- Make sure that the message has been sent.

Phone call and VideoConsultation

- Identify yourself and the citizen.
- Explain how the visit will be approached.
- Inform the citizen that the session is private and confidential.
- Confirm that the citizen has privacy to carry out the visit.
- Confirm that the citizen has time, battery, coverage and connectivity.
- Conduct the clinical interview with: active listening, empathy and giving clear, easy-to-understand messages adapted to the profile of the citizen.
- Summarize the visit and check that the citizen has understood the information given and the next steps (if necessary).

Citizens must be told to register in **the digital health portal "La Meva Salut" and informed about the services it offers**: agenda, previous appointment, access to the eConsultation and soon to the videoConsultation, consultation and downloading diagnostic test results, reports, medication plan, etc.

La meva /Salut

To register in "La Meva Salut", you can do it through the following form:

- **lamevasalut.gencat.cat/alta**
- or calling to **900 053 723** (Monday to Friday from 8am to 8pm)

3 After the visit

- Send the agreed documentation / information.
- Incorporate the clinical interpretation of the visit into the medical record, just as it is done in a face-to-face visit. Plans the corresponding follow-up tasks.

2020. © Generalitat de Catalunya. Servei Català de la Salut (2.7.2020)

Generalitat de Catalunya

Funded by the European Union though the Structural Reform Support Programme

/Salut

Checklists for phone call, eConsultation and videoConsultation

This sheet is addressed to the care management and IT managers of health centers, and aims to provide a list of general recommendations of the main aspects to be considered in the use of non-face-to-face care channels.

Non-face-to-face protocols

- Define protocols with the typology of visits and the criteria for the use of non-face-to-face channels (phone calls, eConsultation and videoConsultation).
- Develop specific criteria for inclusion / exclusion of citizens for non-presential care.
- Establish registration circuits of the information collected during a non-face-to-face visit, as well as the mechanisms for registration of the non-face-to-face activity.
- Define the information to be provided to the citizen before and during an off-site consultation.

Organization and competences

- Define protocols for non-face-to-face care and the roles of the different professional profiles (leadership, administrative management, information systems, incident management, evaluation of results, experience and satisfaction, etc.)
- Integrate the agendas of the professionals with the non-face-to-face channels.
- Define protocols for recording non-face-to-face activity.
- Have a training plan and guidelines for professionals on the use of non-face-to-face channels

Channels and requirements

- Have hardware in good condition and supervised by the IT departments (cameras, microphones, laptops, tablets, smart phones).
- Ensure secure access to software for the use of non-face-to-face channels, and integrated with the center's information systems.
- Integrate the non-face-to-face channels with the medical record systems.

Legal scope and data protection

- Have the informed consent of the citizen, at least the verbal consent, which includes the channels of non-face-to-face care.
- The information regarding data protection is in the section on privacy policy and data protection and in the terms of use of the corresponding non-face-to-face channel.
- Have manuals of conduct for professionals to ensure the quality of the visit and the rights of citizens to privacy and data protection.

Key Aspects

/ Protocols
It is important to establish clear and consensual protocols with the care management and IT departments for the use and integration of the channels in daily practice.

/ Change Management
It is essential to consider the perspective of the professional and the citizens in the change management towards a model that combines face-to-face and non face-to-face care. It is recommended to identify people who support the change.

/ Training and channels
It is necessary to guarantee a correct training by the health center personnel in the use of the different channels of non-face-to-face care.

Channels

/ Equipment
It is necessary to ensure the availability of adequate material for the use of the non-face-to-face care channels. It is necessary to avoid the use of devices that are not provided by the same health centers.

/Software
it is necessary to maintain the programs of non-face-to-face care updated.

2020. © Generalitat de Catalunya. Servei Català de la Salut (2.7.2020)

Generalitat de Catalunya

Funded by the European Union though the Structural Reform Support Programme

/Salut

Supplementary Tables

Table S1. Characteristics of the information stored in the shared electronic health record of Catalonia.

Type of information	Structured	Observations	Standard	Inclusion date	Total volume of instances	2023 volume
Documents	No	Clinical reports, imaging-related reports, complementary test reports, laboratory reports, and pathological anatomy.	SNOMED-CT[a] (only the document type)	July 23, 2008	333,058,853	28,961,626
Medical images	No	WS[b] to publish the medical images taken at the centers and previously registered on SIMDCAT[c]	SERAM[d] or SEMNIM[e]	August 8, 2008	103,640,491	9,874,742
Pathological anatomy results	Yes	Data of the samples and results (conclusions)	SNOMED-CT	February 11, 2013	6,787,863	780,580
Clinical laboratory results	Yes	Laboratory determination data	LOINC[f]	May 7, 2014	72,634,412	8,966,224

Immunizations	Yes	Data on administered vaccines	SNOMED-CT	January 25, 2009	131,342,725	6,953,374
Diagnostics	Yes	Health problems and allergies identified by health centers	ICD-9[g] and ICD-10[h]	November 11, 2009	3,959,522	74,802
Chronic markers	Yes	Identification of patients with chronic illnesses (PCC[i]/MACA[j])	Proprietary	July 14, 2008	408,001	113,723
Spirometry	Yes	Collection of spirometry test data	SNOMED-CT	September 12, 2013	820,842	67,947
Cancer screening	Yes	Data from breast and colon/rectal cancer screenings	SNOMED-CT	July 20, 2023	155,474	155,474
Agendas	Yes	Information on visits to health centers planned by citizens	Proprietary	—[k]	—[k]	—[k]
Clinical parameters	Yes	Data on clinical variables or functional assessment scales	SNOMED-CT	October 3, 2017	276,471,118	35,452,776
Clinical course	Yes/no	Semistructured information—the content of each	Proprietary	October 26, 2017	121,549,421	18,944,002

		section of a clinical course is unstructured information				
DAIA[l] warnings	Yes	Nonpersistent information in the HC3[m] on alerts of files opened by the DGAIA[n]—RSA[o]	Proprietary	February 23, 2009	8194	1099
Organ donation notice	Yes	Nonpersistent information in the HC3 on the official register of organ donors—RSA	Proprietary	May 23, 2016	349,843	31,098
Notice of last wills	Yes	Nonpersistent information in the HC3 official register—RSA	Proprietary	January 2, 2012	153,753	22,054
Social consent	Yes	Information on citizens who have given consent to exchange health and social data	Proprietary	January 26, 2017	21,350	2363

Social data	No	Nonpersistent information in the HC3 of social data of the city councils	Proprietary	—[p]	—[p]	—[p]

[a]SNOMED-CT: Systematized Nomenclature of Medicine - Clinical Terms.

[b]WS: Web services.

[c]SIMDCAT: Digital Medical Imaging System of Catalonia.

[d]SERAM: *Sociedad Española de Radiología Médica* (Spanish Society of Medical Radiology).

[e]SEMNIM: *Sociedad Española de Medicina Nuclear e Imagen Molecular* (Spanish Society of Nuclear Medicine and Molecular Imaging).

[f]LOINC: Local Observation Identifiers Names and Codes.

[g]ICD-9: International Classification of Diseases, Ninth Revision.

[h]ICD-10: International Classification of Diseases, 10th Revision.

[i]PCC: *Pacient Crònic Complex* (Complex Chronic Patients)

[j]MACA: *Malaltia Crònica Avançada* (Advanced Chronic Disease).

[k]Not available—the historical data are not stored.

[l]DAIA: *Declaración de Actividades de Interés* (Declaration of Activities of Interest)

[m]HC3: *Història Clínica Compartida de Catalunya* (Shared Electronic Health Record of Catalonia).

[n]DGAIA: *Direcció General d'Atenció a la Infància i l'Adolescència* (General Directorate for Child and Adolescent Care)

[o]RSA: *Recompte de Serveis Assistencials* (Count of Healthcare Services)

[p]Not available—it is an exchange of data; there is no record regarding the number of requests

Table S2. Current storage of the SIMDCAT federated platform according to diagnostic imaging techniques (from inception and until 31/12/2023)

**Diagnostic Imaging Technique**	**Total**	
	Number of studies	Volume (GB)
Computed Radiography	36,676,932	411,680.41
Digital Radiography	22,907,474	259,682.67
Ultrasound	15,933,834	634,394.23
Electrocardiography	12,768,938	4,143.45
Computed Tomography	9,666,140	1,489,050.66
Mammography	5,023,883	354,210.57
Magnetic Resonance	4,664,926	389,807.17
External-camera Photography	1,704,032	28,766.93
Secondary Capture	1,551,937	36,242.46
Endoscopy	1,283,128	16,725.50
Nuclear Medicine	1,027,777	8,750.36
Other	981,211	14,201.85
Panoramic X-Ray	859,094	4,464.15
Fluoroscopy	639,584	23,695.14
X-Ray Angiography	616,858	118,270.14
Ophthalmic Tomography	477,406	11,364.07
Ophthalmic Visual Field	257,317	38.75
SR Document (Structured Report)	227,976	39.68
Positron emission tomography	177,965	16,684.57
Ophthalmic Photography	124,119	4,728.03
Visible Light	100,762	1,373.34
Presentation State	61,457	9.44
Document	58,971	22.69
Intra-oral Radiography	57,152	40.65
Ophthalmic Axial Measurements	8,844	36.49
Intraocular Lens Data	7,555	2.18
Digital microscopy	6,634	75.47
Optical Coherence Tomography	6,512	669.75
Ophthalmic Mapping	6,475	32.86
Bone Mineral Densitometry	4,766	12.16
Segmentation	3,935	835.99
Key Object Selection	2,838	0.08
Intravascular Ultrasound	1,197	176.95
Keratometry	42	0.01
General Microscopy	20	0.18
**TOTAL**	**117,897,691**	**3,830,229.01**

Table S3. ePROMs implemented within the Catalan Healthcare system.

ePROM	Speciality	Implementation date
EQ-5D-5L, EuroQol 5 Dimensions 5 Levels [1,2]	General/Family Medicine	08/03/2022
GAD-7, generalized anxiety disorder scale [3,4]	Psychiatry	15/11/2022
PHQ-9, depression scale [5–7]	Psychiatry	15/11/2022
IPSS, International Prostate Symptom Score [8,9]	Urology	15/11/2022
AUDIT, Alcohol Use Disorders Identification Test (pregnancy) [10–18]	Obstetrics	30/06/2023
Roland-Morris, lumbar pain disability assessment scale [19,20]	Rehabilitation	30/06/2023
WEMWBS, Warwick-Edinburgh Mental Well-being Scale [21,22]	General/Family Medicine	30/06/2023
NPQ, Northwick Park Neck Pain Questionnaire [23,24]	Rehabilitation	30/06/2023
SPADI, Shoulder Pain And Disability Index [25–27]	Rehabilitation	30/06/2023
WOMAC, knee and hip osteoarthritis questionnaire [28,29]	Rehabilitation	30/06/2023
IPAQ, International Physical Activity Questionnaire [30]	General/Family Medicine	30/06/2023
PURE-4, Psoriatic arthritis UnclutteRed screening Evaluation [31–33]	Rheumatology	30/06/2023
DDS17, Diabetes Distress Scale [34]	Endocrinology	30/06/2023
IIEF-5, International Index of Erectile Dysfunction-5 [35,36]	Urology	30/06/2023
Stoma-QoL, quality of life questionnaire for ostomy patients [37]	General and Digestive Surgery	28/09/2023
DHI, Dizziness Handicap Inventory [38,39]	Neurology	28/09/2023
Edinburgh Depression Scale (postpartum) [40,41]	Obstetrics	28/09/2023

Edinburgh Depression Scale (pregnancy) [40,42]	Obstetrics	28/09/2023
SF-MPQ, McGill Pain Questionnaire [43–46]	General/Family Medicine	28/09/2023
DME-E, emotional distress scale of patients with advanced illness [47,48]	General/Family Medicine	28/09/2023
DME-C, emotional distress scale of the primary caregiver of patients with advanced illness [49]	General/Family Medicine	28/09/2023
ESAS-r, Edmonton Symptom Assessment System-revised questionnaire [50,51]	Oncology	28/09/2023
EPIC-26, Expanded Prostate Cancer Index Composite [52,53]	Urology	13/12/2023
GDS-5, Geriatric Depression Scale (5 questions) [54–56]	Geriatrics	13/12/2023
ARMS, Adherence to Refill and Medication Scale [57,58]	General/Family Medicine	13/12/2023
PREDIMED, adherence to Mediterranean diet questionnaire (*PREvención con DIeta MEDiterránea*) [59]	General/Family Medicine	13/12/2023
SF-ICIQ, Short Form International Consultation on Incontinence Questionnaire [60,61]	General/Family Medicine	13/12/2023
HFS, Hypoglycaemia Fear Survey [62,63]	Endocrinology	13/12/2023
AUDIT, alcohol use disorders identification test (non-pregnant men/women) [11,13,16,18]	General/Family Medicine	13/12/2023
GDS-15, Geriatric Depression Scale (15 questions) [64,65]	Geriatrics	19/03/2024
SWEMWBS, Short Warwick-Edinburgh Mental Well-being Scale [22,66,67]	General/Family Medicine	19/03/2024
AUDIT-C, Alcohol Use Disorders Identification Test – Consumption (general population) [13,16,18,68]	General/Family Medicine	19/03/2024
AUDIT-C, Alcohol Use Disorders Identification Test – Consumption (pregnancy) [13,14,16–18,68]	Obstetrics	19/03/2024

FTND, Fagerström Test for Nicotine Dependence [69–71]	General/Family Medicine	19/03/2024
FTND, Fagerström Test for Nicotine Dependence (short) [69–72]	General/Family Medicine	19/03/2024
OSSS-3, Oslo Social Support Scale [73]	General/Family Medicine	19/03/2024
PVS, Partner Violence Screen scale [74,75]	General/Family Medicine	19/03/2024

Supplementary References

1. Janssen MF, Birnie E, Haagsma JA, Bonsel GJ. Comparing the standard EQ-5D three-level system with a five-level version. Value in Health 2008;11(2):275–284. PMID:18380640

2. Hernandez G, Garin O, Pardo Y, Vilagut G, Pont À, Suárez M, Neira M, Rajmil L, Gorostiza I, Ramallo-Fariña Y, Cabases J, Alonso J, Ferrer M. Validity of the EQ–5D–5L and reference norms for the Spanish population. Quality of Life Research Springer International Publishing; 2018 Sep 1;27(9):2337–2348. PMID:29767329

3. Spitzer RL, Kroenke K, Williams JBW, Löwe B. A brief measure for assessing generalized anxiety disorder: The GAD-7. Arch Intern Med American Medical Association; 2006 May 22;166(10):1092–1097. PMID:16717171

4. García-Campayo J, Zamorano E, Ruiz MA, Pardo A, Pérez-Páramo M, López-Gómez V, Freire O, Rejas J. Cultural adaptation into Spanish of the generalized anxiety disorder-7 (GAD-7) scale as a screening tool. Health Qual Life Outcomes BioMed Central; 2010 Jan 20;8(1):1–11. PMID:20089179

5. Kroenke K, Spitzer RL, Williams JBW. The PHQ-9: Validity of a brief depression severity measure. J Gen Intern Med Springer; 2001;16(9):606–613. PMID:11556941

6. Kroenke K, Spitzer RL. The PHQ-9: A new depression diagnostic and severity measure. Psychiatr Ann. Slack Incorporated; 2002. p. 509–515. doi: 10.3928/0048-5713-20020901-06

7. Diez-Quevedo C, Rangil T, Sanchez-Planell L, Kroenke K, Spitzer RL. Validation and utility of the patient health questionnaire in diagnosing mental disorders in 1003 general hospital Spanish inpatients. Psychosom Med Psychosom Med; 2001;63(4):679–686. PMID:11485122

8. Barry MJ, Fowler FJ, O'Leary MP, Bruskewitz RC, Holtgrewe HL, Mebust WK, Cockett ATK, Blaivas JG, Wein AJ. The American Urological Association symptom index for benign prostatic hyperplasia. Journal of Urology Wolters KluwerPhiladelphia, PA; 1992;148(5 I):1549–1557. PMID:1279218

9. Badía X, García-Losa M, Dal-Ré R, Carballido J, Serra M. Validation of a harmonized Spanish version of the IPSS: Evidence of equivalence with the original American scale. Urology Elsevier; 1998 Oct 1;52(4):614–620. PMID:9763080

10. Saunders JB, Aasland OG, Amundsen A, Grant M. Alcohol consumption and related problems among primary health care patients: WHO Collaborative Project on Early Detection of Persons with Harmful Alcohol Consumption—I. Addiction John Wiley & Sons, Ltd; 1993 Mar 1;88(3):349–362. PMID:8461852

11. Saunders JB, Aasland OG, Babor TF, De La Fuente JR, Grant M. Development of the Alcohol Use Disorders Identification Test (AUDIT): WHO Collaborative Project on Early Detection of Persons with Harmful Alcohol Consumption-II. Addiction John Wiley & Sons, Ltd; 1993 Jun 1;88(6):791–804. PMID:8329970

12. Isaacson JH, Butler R, Zacharke M, Tzelepis A. Screening with the alcohol use disorders identification test (AUDIT) in an inner-city population. J Gen Intern Med Springer; 1994;9(10):550–553. PMID:7823225

13. Rubio Valladolid G, Bermejo Vicedo J, Caballero Sánchez-Serrano MC, Santo-Domingo Carrasco J. Validation of the Alcohol Use Disorders Identification Test (AUDIT) in primary care. Rev Clin Esp Spain; 1998 Jan;198(1):11–14. PMID:9534342

14. Bradley KA, Boyd-Wickizer J, Powell SH, Burman ML. Alcohol Screening Questionnaires in Women: A Critical Review. JAMA American Medical Association; 1998 Jul 8;280(2):166–171. PMID:9669791

15. Bradley KA, Bush KR, McDonell MB, Malone T, Fihn SD. Screening for problem drinking: Comparison of CAGE and AUDIT. J Gen Intern Med Springer New York LLC; 1998;13(6):379–389. PMID:9669567

16. Contel Guillamon M, Gual Sole A, Colom Farran J. Alcohol use disorders identification test (AUDIT): Translation and validation of Catalan and Spanish. Adicciones Edita Socidrogalcohol; 1999 Dec 15;11(4):337–347. doi: 10.20882/adicciones.613

17. Dawson DA, Grant BF, Stinson FS, Zhou Y. Effectiveness of the derived Alcohol Use Disorders Identification Test (AUDIT-C) in screening for alcohol use disorders and risk drinking in the US general population. Alcohol Clin Exp Res John Wiley & Sons, Ltd; 2005 May 1;29(5):844–854. PMID:15897730

18. de Torres LAP, Rebollo EM, Ruiz-Moral R, Fernández-García JA, Vega RA, Palomino MM. Diagnostic usefulness of the Alcohol Use Disorders Identification Test (AUDIT) questionnaire for the detection of hazardous drinking and dependence on alcohol among Spanish patients. European Journal of General Practice Taylor & Francis; 2009;15(1):15–21. PMID:19418376

19. Roland M, Morris R. A study of the natural history of back pain. Part I: development of a reliable and sensitive measure of disability in low-back pain. Spine (Phila Pa 1976) Spine (Phila Pa 1976); 1983;8(2):141–144. PMID:6222486

20. Kovacs FM, Llobera J, Gil del Real MT, Abraira V, Gestoso M, Fernández C, Bauza JR, Bauza K, Coll J, Duro E, Gili J, Gómez M, González J, Ibañez P, Jover A, Lázaro P, Llinás M, Mateu C, Mufraggi N, Nicolau C, Olivera MA, Pascual P, Perelló L, Pozo F, Reyes V, Ribot S, Ripoll J, Ripoll J, Rodríguez E. Validation of the spanish version of the Roland-Morris questionnaire. Spine (Phila Pa 1976) Spine (Phila Pa 1976); 2002 Mar 1;27(5):538–542. PMID:11880841

21. Tennant R, Hiller L, Fishwick R, Platt S, Joseph S, Weich S, Parkinson J, Secker J, Stewart-Brown S. The Warwick-Dinburgh mental well-being scale (WEMWBS): Development and UK validation. Health Qual Life Outcomes BioMed Central; 2007 Nov 27;5(1):1–13. PMID:18042300

22. Castellví P, Forero CG, Codony M, Vilagut G, Brugulat P, Medina A, Gabilondo A, Mompart A, Colom J, Tresserras R, Ferrer M, Stewart-Brown S, Alonso J. The Spanish version of the Warwick-Edinburgh Mental Well-Being Scale (WEMWBS) is valid for use in the general population. Quality of Life Research Kluwer Academic Publishers; 2014 Sep 5;23(3):857–868. PMID:24005886

23. Leak AM, Frank AO. The Northwick Park Neck Pain Questionnaire, Devised to Measure Neck Pain and Disability. Rheumatology Oxford Academic; 1994 Dec 1;33(12):1204–1204. doi: 10.1093/RHEUMATOLOGY/33.12.1204

24. González T, Balsa A, Sáinz de Murieta J, Zamorano E, González I, Martin-Mola E. Spanish version of the Northwick Park Neck Pain Questionnaire: reliability and validity. Clin Exp Rheumatol Italy; 2001;19(1):41–46. PMID:11247324

25. Roach KE, Budiman-Mak E, Songsiridej N, Lertratanakul Y. Development of a Shoulder Pain and Disability Index. Arthritis Rheum John Wiley & Sons, Ltd; 1991 Dec 1;4(4):143–149. PMID:11188601

26. Schmidt S, Ferrer M, González M, González N, Valderas JM, Alonso J, Escobar A, Vrotsou K, Montse F, Stefanie S, Olatz G, Gemma V, Angels P, Yolanda P, Gabriela B, Pere C, Carlos GF, Ana R, Virginia B, Ester V, Mireya GD, Sonia R, Oriol C, José María RR. Evaluation of shoulder-specific patient-reported outcome measures: A systematic and standardized comparison of available evidence. J Shoulder Elbow Surg Mosby Inc.; 2014 Mar 1;23(3):434–444. PMID:24406123

27. Membrilla-Mesa MD, Cuesta-Vargas AI, Pozuelo-Calvo R, Tejero-Fernández V, Martín-Martín L, Arroyo-Morales M. Shoulder pain and disability index: Cross cultural validation and evaluation of psychometric properties of the Spanish version. Health Qual Life Outcomes BioMed Central Ltd.; 2015 Dec 21;13(1):1–6. PMID:26690943

28. Bellamy N, Buchanan WW, Goldsmith CH, Campbell J, Stitt LW. Validation study of WOMAC: a health status instrument for measuring clinically important patient relevant outcomes to antirheumatic drug therapy in patients with osteoarthritis of the hip or knee. J Rheumatol Canada; 1988 Dec;15(12):1833–1840. PMID:3068365

29. Escobar A, Quintana JM, Bilbao A, Azkárte J, Güenaga LI. Validation of the Spanish version of the WOMAC questionaire for patients with hip or knee osteoarthritis. Clin Rheumatol Springer; 2002 Nov;21(6):466–471. PMID:12447629

30. Craig CL, Marshall AL, Sjöström M, Bauman AE, Booth ML, Ainsworth BE, Pratt M, Ekelund U, Yngve A, Sallis JF, Oja P. International physical activity questionnaire: 12-Country reliability and validity. Med Sci Sports Exerc 2003 Aug 1;35(8):1381–1395. PMID:12900694

31. Audureau E, Roux F, Lons Danic D, Bagot M, Cantagrel A, Dernis E, Gouyette N, Hilliquin P, Jullien D, Lioté F, Passeron T, A Richard M, Claudepierre P. Psoriatic arthritis screening by the dermatologist: development and first validation of the 'PURE-4 scale.' Journal of the European Academy of Dermatology and Venereology John Wiley & Sons, Ltd; 2018 Nov 1;32(11):1950–1953. PMID:29430720

32. Belinchón I, Queiro R, Salgado-Boquete L, López-Ferrer A, Ferran M, Coto-Segura P, Rivera R, Vidal D, Rodríguez L, de la Cueva P, Guinea G, Martin Vazquez V. Linguistic and Cultural Adaptation to Spanish of the Screening Tool Psoriatic Arthritis UnclutteRed Screening Evaluation (PURE4). Actas Dermosifiliogr Elsevier Doyma; 2020 Oct 1;111(8):655–664. PMID:32401721

33. Romero IB, Ferrer AL, i Farrés MF, Díaz RR, Sarro DV, Fernández-Freire LR, Cueva-Dobao P de la, Juanes JS, Duran VR, Vázquez VM, Labradror LG, Silva RQ. Validación de la versión española del cuestionario PURE-4 para la detección precoz de la artritis psoriásica en pacientes con psoriasis. Actas Dermosifiliogr Elsevier Doyma; 2024 Feb 19; PMID:38382746

34. Polonsky WH, Fisher L, Earles J, Dudl RJ, Lees J, Mullan J, Jackson RA. Assessing psychosocial distress in diabetes: Development of the Diabetes Distress Scale. Diabetes Care American Diabetes Association; 2005 Mar 1;28(3):626–631. PMID:15735199

35. Rhoden EL, Telöken C, Sogari PR, Vargas Souto CA. The use of the simplified International Index of Erectile Function (IIEF-5) as a diagnostic tool to study the prevalence of erectile dysfunction. Int J Impot Res Nature Publishing Group; 2002 Aug 5;14(4):245–250. PMID:12152112

36. Lizarraga-Limousin R, Medrano-Sánchez EM, Díaz-Mohedo E, Vergara-De-carlos L. Spanish Cross-Cultural Validation of the Electronic Version of the International Index of Erectile Function-5 (IIEF-5). Int J Environ Res Public Health Multidisciplinary Digital Publishing Institute; 2022 Mar 7;19(5):3115. PMID:35270819

37. Prieto L, Thorsen H, Juul K. Development and validation of a quality of life questionnaire for patients with colostomy of ileostomy. Health Qual Life Outcomes BioMed Central; 2005 Oct 12;3(1):1–10. PMID:16219109

38. Jacobson GP, Newman CW. The Development of the Dizziness Handicap Inventory. Arch Otolaryngol Head Neck Surg American Medical Association; 1990 Apr 1;116(4):424–427. PMID:2317323

39. Pérez N, Garmendia I, Martín E, García-Tapia R. Cultural adaptation of 2 questionnaires for health measurement in patients with vertigo. Acta Otorrinolaringol Esp Spain; 2000 Oct;51(7):572–580. PMID:11270034

40. Cox JL, Holden JM, Sagovsky R. Detection of Postnatal Depression: Development of the 10-item Edinburgh Postnatal Depression scale. British Journal of Psychiatry Cambridge University Press; 1987;150(JUNE):782–786. PMID:3651732

41. Garcia-Esteve L, Ascaso C, Ojuel J, Navarro P. Validation of the Edinburgh Postnatal Depression Scale (EPDS) in Spanish mothers. J Affect Disord Elsevier; 2003 Jun 1;75(1):71–76. PMID:12781353

42. Vázquez MB, Míguez MC. Validation of the Edinburgh postnatal depression scale as a screening tool for depression in Spanish pregnant women. J Affect Disord Elsevier; 2019 Mar 1;246:515–521. PMID:30599376

43. Melzack R. The short-form McGill Pain Questionnaire. Pain United States; 1987 Aug;30(2):191–197. PMID:3670870

44. Lázaro C, Bosch F, Torrubia R, Banos J-E. The development of a Spanish questionnaire for assessing pain: Preliminary data concerning reliability and validity. European Journal of Psychological Assessment 1994;10:145–151.

45. Masedo AI, Esteve R. Some empirical evidence regarding the validity of the Spanish Version of the McGill Pain Questionnaire (MPQ-SV). Pain 2000 Apr 1;85(3):451–456. PMID:10781918

46. Lázaro C, Caseras X, Whizar-Lugo VM, Wenk R, Baldioceda F, Bernal R, Ovalle A, Torrubia R, Baños JE. Psychometric properties of a Spanish version of the Mcgill Pain Questionnaire in several Spanish-speaking countries. Clinical Journal of Pain Clin J Pain; 2001;17(4):365–374. PMID:11783818

47. Maté J, Mateo D, Bayés R, Bernaus M, Casas C, González-Barboteo J, Limonero JT, López-Postigo M, Sirgo A, Viel S. Elaboración y propuesta de un instrumento para la detección de malestar emocional en enfermos al final de la vida. Psicooncologia (Pozuelo de Alarcon) 2009;6(2–3):499–506. Available from: https://revistas.ucm.es/index.php/PSIC/article/view/PSIC0909220507A [accessed Mar 25, 2024]

48. Limonero JT, Mateo D, Maté-Méndez J, González-Barboteo J, Bayés R, Bernaus M, Casas C, López M, Sirgo A, Viel S. Evaluación de las propiedades psicométricas del cuestionario de Detección de Malestar Emocional (DME) en pacientes oncológicos. Gac Sanit Sociedad Española de Salud Pública y Administración Sanitaria (SESPAS); 2012;26(2):145–152. PMID:22033008

49. Limonero JT, Maté J, Mateo D, González-Barboteo J, Bayés R, Bernaus M, Casas C, López M, Sirgo A, Viel S, Sánchez C, Gómez-Romero MJ, Álvarez-Moleiro M, Tomás-Sábado J. Development of the DME-C: A scale for detecting emotional distress in primary caregivers of patients living with advanced illness or approaching end of their lives. Ansiedad y Estres Elsevier; 2016 Jul 1;22(2–3):104–109. doi: 10.1016/j.anyes.2016.09.001

50. Watanabe SM, Nekolaichuk C, Beaumont C, Johnson L, Myers J, Strasser F. A multicenter study comparing two numerical versions of the Edmonton symptom assessment system in palliative care patients. J Pain Symptom Manage Elsevier; 2011 Feb 1;41(2):456–468. PMID:20832987

51. Carvajal A, Hribernik N, Duarte E, Sanz-Rubiales A, Centeno C. The Spanish Version of the Edmonton Symptom Assessment System-Revised (ESAS-r): First Psychometric Analysis Involving Patients with Advanced Cancer. J Pain Symptom Manage Elsevier; 2013 Jan 1;45(1):129–136. PMID:22926088

52. Szymanski KM, Wei JT, Dunn RL, Sanda MG. Development and validation of an abbreviated version of the expanded prostate cancer index composite instrument for measuring health-related quality of life among prostate cancer survivors. Urology Elsevier; 2010 Nov 1;76(5):1245–1250. PMID:20350762

53. Zamora V, Garin O, Suárez JF, Jové J, Castells M, Ferrer F, Gutiérrez C, Guedea F, Boladeras A, Fumadó L, Roselló A, Pastor J, Samper P, Pont À, Ferrer M. Psychometric validation of the Spanish version of the Expanded Prostate Cancer Index Composite-26. World J Urol Springer Science and Business Media Deutschland GmbH; 2023 Dec 1;41(12):3511–3518. PMID:37947846

54. Hoyl MT, Alessi CA, Harker JO, Josephson KR, Pietruszka FM, Koelfgen M, Mervis JR, Fitten LJ, Rubenstein LZ. Development and testing of a five-item version of the geriatric depression scale. J Am Geriatr Soc John Wiley & Sons, Ltd; 1999 Jul 1;47(7):873–878. PMID:10404935

55. De Dios del Valle R, Hernández Sánchez AM, Rexach Cano LI, Cruz Jentoft AJ. Validación de una versión de cinco ítems de la Escala de Depresión Geriátrica de Yesavage en población española. Rev Esp Geriatr Gerontol Elsevier Doyma; 2001 Jan 1;36(5):276–280. doi: 10.1016/s0211-139x(01)74736-1

56. Izal M, Montorio I, Nuevo R, Pérez-Rojo G. Comparación de la sensibilidad y la especificidad entre diferentes versiones de la Escala de Depresión Geriátrica. Rev Esp

Geriatr Gerontol Elsevier Doyma; 2007 Jul 1;42(4):227–232. doi: 10.1016/S0211-139X(07)73555-2

57. Kripalani S, Risser J, Gatti ME, Jacobson TA. Development and Evaluation of the Adherence to Refills and Medications Scale (ARMS) among Low-Literacy Patients with Chronic Disease. 2009; doi: 10.1111/j.1524-4733.2008.00400.x

58. González-Bueno J, Calvo-Cidoncha E, Sevilla-Sánchez D, Espaulella-Panicot J, Codina-Jané C, Santos-Ramos B. Traducción y adaptación transcultural al español del cuestionario ARMS para la medida de la adherencia en pacientes pluripatológicos. Aten Primaria Elsevier Doyma; 2017 Oct 1;49(8):459–464. PMID:28390732

59. Martínez-González MA, García-Arellano A, Toledo E, Salas-Salvadó J, Buil-Cosiales P, Corella D, Covas MI, Schröder H, Arós F, Gómez-Gracia E, Fiol M, Ruiz-Gutiérrez V, Lapetra J, Lamuela-Raventos RM, Serra-Majem L, Pintó X, Muñoz MA, Wärnberg J, Ros E, Estruch R. A 14-item mediterranean diet assessment tool and obesity indexes among high-risk subjects: The PREDIMED trial. PLoS One Public Library of Science; 2012 Aug 14;7(8):e43134. PMID:22905215

60. Avery K, Donovan J, Peters TJ, Shaw C, Gotoh M, Abrams P. ICIQ: A brief and robust measure for evaluating the symptoms and impact of urinary incontinence. Neurourol Urodyn. John Wiley & Sons, Ltd; 2004. p. 322–330. PMID:15227649

61. Espuña Pons M, Rebollo Álvarez P, Puig Clota M. Validación de la versión española del International Consultation on Incontinence Questionnaire-Short Form. Un cuestionario para evaluar la incontinencia urinaria. Med Clin (Barc) Elsevier Doyma; 2004 Jan 1;122(8):288–292. doi: 10.1016/s0025-7753(04)74212-8

62. Cox DJ, Irvine A, Gonder-Frederick L, Nowacek G, Butterfield J. Fear of hypoglycemia: Quantification, validation, and utilization. Diabetes Care American Diabetes Association; 1987 Sep 1;10(5):617–621. PMID:3677982

63. Tasende C, Rubio JA, Álvarez J. Spanish translation, adaptation and validation of the Hypoglycemia Fear Survey in adults with type 1 diabetes in the Community of Madrid. Endocrinología, Diabetes y Nutrición (English ed) Elsevier Doyma; 2018 May 1;65(5):287–296. doi: 10.1016/j.endien.2018.05.002

64. Yesavage JA, Sheikh JI. Geriatric Depression Scale (GDS). Clin Gerontol Routledge; 1986 Nov 18;5(1–2):165–173. doi: 10.1300/J018v05n01_09

65. Martínez J, Onis C, Duenas R, Colomer A, Aguado C, Luque R. The Spanish version of the Yesavage abbreviated questionnaire (GDS) to screen depressive dysfunctions in patients older than 65 years. [Spanish] TT - Version espanola del cuestionario de Yesavage abreviado (GDS) para el despistaje de depresion en mayores. MEDIFAM - Revista de Medicina Familiar y Comunitaria Arán Ediciones, S. L.; 2002;12(10):620–630. Available from: https://scielo.isciii.es/scielo.php?script=sci_arttext&pid=S1131-57682002001000003&lng=en&nrm=iso&tlng=en [accessed Mar 25, 2024]

66. Koushede V, Lasgaard M, Hinrichsen C, Meilstrup C, Nielsen L, Rayce SB, Torres-Sahli M, Gudmundsdottir DG, Stewart-Brown S, Santini ZI. Measuring mental well-being in Denmark: Validation of the original and short version of the Warwick-Edinburgh mental

well-being scale (WEMWBS and SWEMWBS) and cross-cultural comparison across four European settings. Psychiatry Res Elsevier; 2019 Jan 1;271:502–509. PMID:30551082

67. Shah N, Cader M, Andrews B, McCabe R, Stewart-Brown SL. Short Warwick-Edinburgh Mental Well-being Scale (SWEMWBS): performance in a clinical sample in relation to PHQ-9 and GAD-7. Health Qual Life Outcomes BMC; 2021 Dec 1;19(1). PMID:34819104

68. Bush K, Kivlahan DR, McDonell MB, Fihn SD, Bradley KA. The AUDIT alcohol consumption questions (AUDIT-C): an effective brief screening test for problem drinking. Ambulatory Care Quality Improvement Project (ACQUIP). Alcohol Use Disorders Identification Test. Arch Intern Med Arch Intern Med; 1998 Sep 14;158(16):1789–1795. PMID:9738608

69. Becoña E, Vázquez FL. The Fagerström Test for Nicotine Dependence in a Spanish sample. Psychol Rep SAGE PublicationsSage CA: Los Angeles, CA; 1998 Dec 1;83(3 PART 2):1455–1458. PMID:10079737

70. HEATHERTON TF, KOZLOWSKI LT, FRECKER RC, FAGERSTROM K -O. The Fagerström Test for Nicotine Dependence: a revision of the Fagerstrom Tolerance Questionnaire. Br J Addict John Wiley & Sons, Ltd; 1991 Sep 1;86(9):1119–1127. PMID:1932883

71. Roa-Cubaque MA, Parada-Sierra ZE, Albarracín-Guevara Y, Alba-Castro E, Aunta-Piracon M, Ortiz-León M. Validación del test de Fagerström para adicción a la nicotina (FTND). Revista Investigación en Salud Universidad de Boyacá Universidad de Boyaca; 2016 Dec 1;3(2):161–175. doi: 10.24267/23897325.185

72. John U, Meyer C, Schumann A, Hapke U, Rumpf HJ, Adam C, Alte D, Lüdemann J. A short form of the Fagerström Test for Nicotine Dependence and the Heaviness of Smoking Index in two adult population samples. Addictive Behaviors Pergamon; 2004 Aug 1;29(6):1207–1212. PMID:15236824

73. Kocalevent RD, Berg L, Beutel ME, Hinz A, Zenger M, Härter M, Nater U, Brähler E. Social support in the general population: Standardization of the Oslo social support scale (OSSS-3). BMC Psychol BMC; 2018 Jul 17;6(1). PMID:30016997

74. Feldhaus KM, Koziol-McLain J, Amsbury HL, Norton llena M, Lowenstein SR, Abbott JT. Accuracy of 3 Brief Screening Questions for Detecting Partner Violence in the Emergency Department. JAMA 1997 May 7;277(17):1357–1361. doi: 10.1001/jama.1997.03540410035027

75. Garcia-Esteve L, Torres A, Navarro P, Ascaso C, Imaz ML, Herreras Z, Valdés M. Validación y comparación de cuatro instrumentos para la detección de la violencia de pareja en el ámbito sanitario. Med Clin (Barc) Elsevier Doyma; 2011 Oct 8;137(9):390–397. PMID:21757210
